# Myxobacteria of the *Cystobacterineae* Suborder Are Producers of New Vitamin K_2_ Derived Myxoquinones

**DOI:** 10.3390/microorganisms10030534

**Published:** 2022-02-28

**Authors:** Fabian Panter, Alexander Popoff, Ronald Garcia, Daniel Krug, Rolf Müller

**Affiliations:** 1 Department of Microbial Natural Products, Helmholtz-Institute for Pharmaceutical Research Saarland (HIPS), Helmholtz Centre for Infection Research (HZI), Saarland University, Campus E8 1, 66123 Saarbrücken, Germany; fabian.panter@helmholtz-hips.de (F.P.); alexander.popoff@helmholtz-hips.de (A.P.); ronald.garcia@helmholtz-hips.de (R.G.); daniel.krug@helmholtz-hips.de (D.K.); 2 German Centre for Infection Research (DZIF), Partner Site Hannover-Braunschweig, 38124 Braunschweig, Germany; 3 Helmholtz International Lab for Anti-Infectives, Campus E8 1, 66123 Saarbrücken, Germany

**Keywords:** vitamin K, menaquinone, myxobacteria, terpenes, structure elucidation

## Abstract

Vitamin K is an essential, lipid soluble vitamin that plays an important role in the human blood coagulation cascade as well as in the life cycle of bacteria and plants. In this study, we report the isolation and structure elucidation of unprecedented polyhydroxylated menaquinone variants named myxoquinones that are produced by myxobacteria and structurally belong to the Vitamin K family. We analyze the occurrence of myxoquinones across an LC-MS data collection from myxobacterial extracts and shed light on the distribution of myxoquinone-type biosynthetic gene clusters among publicly available myxobacterial genomes. Our findings indicate that myxoquinones are specifically produced by strains of the *Cystobacterineae* suborder within myxobacteria. Furthermore, bioinformatic analysis of the matching gene clusters allowed us to propose a biosynthetic model for myxoquinone formation. Due to their increased water-solubility, the myxoquinones could be a suitable starting point for the development of a better bioavailable treatment of vitamin K deficiency.

## 1. Introduction

**Figure 1:**
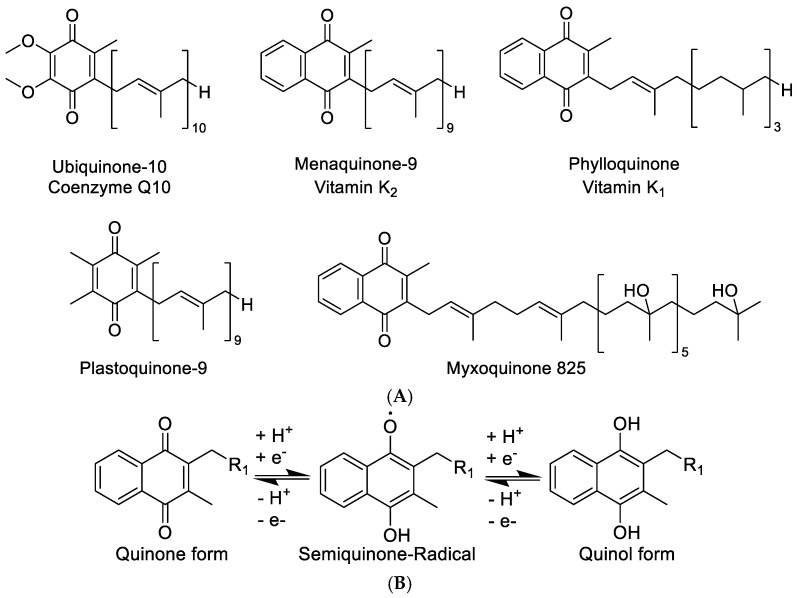
(**A**) Structure overview of ubiquinone, menaquinone, phylloquinone, plastoquinone, and the new myxoquinone-825. (**B**) Quinone and quinol form of naphthoquinone type metabolites.

Vitamin K belongs to the lipid soluble vitamins that are essential to the human diet. Vitamin K deficiency can lead to possibly fatal severe hemorrhaging, as it plays an important role in the blood coagulation cascade. In nature, two distinct sources are classically seen to provide vitamin K, namely, the plant chloroplasts, where it exists as phylloquinone, or vitamin K_1_ and bacterial cells, where it can be found as menaquinone or vitamin K_2_. Recent results in studying the role of Vitamin K in bacteria regarding host–pathogen interactions in algal species have shown that the transfer of the Vitamin K pathway to algae, and by extension to plants, may even have occurred by heterologous gene transfer via intracellular pathogens of the Chlamydiales order [1]. Vitamin K is one of the essential vitamins for humans. Vitamin K deficiency leads to impairment of blood coagulation, which results in hemorrhaging and—in extreme cases—the death of the individual. It was understood very early on that blood clotting, or blood coagulation based on Vitamin K deficiencies, could be cured by supplementing diets with either fermented foods or the green part of plants [2]. This finding is readily explained as fermented foods contain menaquinone of bacterial origin produced during fermentation, while the plant chloroplasts contain the major part of the plantal phylloquinone (Figure 1A). Although the role of phylloquinone in plants is not completely understood, it mainly occurs in the plantal thylakoid membrane taking part in the electron transport chain of photosynthesis. Like the more well-known plastoquinone, phylloquinone seems to play a role in buffering the single electron stream coming off the photosystems in the chloroplasts by storing single electrons as a semi-stable phylloquinone radical (Figure 1B) [3,4]. It therefore possibly acts as an actuator that modulates the single electron stream coming from the photosystem II to the cytochrome b_6_f, similar to the much wider known plastoquinone [5]. Like benzoquinones, molecules featuring a naphthoquinone moiety such as the vitamin K family are able to change between two redox states, namely, the quinone and the quinol form, and store redox potential in that way (Figure 1B) [6]. In addition, 1,4-naphthoquinone type structures stabilize radical electrons in their core as a naphthosemiquinone radical, which enables them to take part in redox reactions requiring subsequent transfer of single electrons [7].

A wide range of bacteria including the biotechnologically relevant species *Escherichia coli* and *Bacillus subtilis* are well-known producers of the Vitamin K_1_ analogue menaquinone [8,9]. Actinobacteria that are mainly used for the biotechnological production of secondary metabolites—among them many antibiotics or anti-cancer agents—are also able to produce a variety of Vitamin K related quinones [10]. For example, members of the actinobacterial genus *Nocardia* are able to produce distinct and unique Vitamin K derivatives as exemplified by the cyclized tail group variants of Vitamin K_2_ produced by the strain *Nocardia brasiliensis* (DSM 43009) [11]. Recently, the spectrum of Vitamin K_2_ derivatives has been thoroughly investigated during attempts to increase biotechnological yields of Vitamin K_2_ for future process development but classical Vitamin K_2_ analogues that are uncovered here typically vary in the length of their terpene side chain ranging from menaquinone 7 to menaquinone 11 [12].

In this work, we describe the myxoquinones, a group of novel Vitamin K_2_-derived myxobacterial metabolites, elaborate on their biosynthetic origin and analyze their distribution among myxobacteria. Our findings lead us to hypothesize that the potential biological role of the myxoquinones in myxobacteria of the *Cystobacerinae* suborder is likely comparable to other quinones involved in biological redox processes.

## 2. Materials and Methods

### 2.1. Cultivation of Myxobacteria

All myxobacterial cultures were grown in 300 mL shake flasks containing 50 mL of VY/2, VY, PYGS, or CyHv3 medium (Tables S1–S4) for both *Corallococcus* sp. MCy9049 and *Myxococcaceae* sp. MCy9003. Following inoculation with 1 mL pre-culture the medium was supplemented with 2% of sterile XAD-16 adsorber resin (Sigma Aldrich, Taufkirchen, Germany) suspension in water to bind metabolites from the culture medium. Small scale cultures were grown for 10–12 days until the fermentation medium had cleared up except for the myxobacterial biofilm clumps and XAD-16 resin particles. After fermentation the cultures were pelleted in a 50 mL tubes at 6000 rcf for 10 min using a table centrifuge (Eppendorf) and stored at −20 °C until further use. The myxobacterial strains were kept in agar culture for storage for timespans of a few days. The agar media used in this case were VY/2 agar, prepared by adding 14 g/L agarose (BD) to VY/2 medium before autoclaving.

### 2.2. Extraction Procedure

Frozen cell pellets were transferred into 100 mL Erlenmeyer flasks and a magnetic stirrer was added. Then, 50 mL of acetone (Fluka analytical grade, redistilled in house) was added onto the pellet and the mixture was stirred for 60 min. The acetone extract was left to settle in order to sediment cell debris and XAD resin for a second extraction step. The supernatant was filtered with a 125-micron-folded filter keeping cell pellet and XAD-16 resin in the Erlenmeyer flask. The residual pellet and XAD-16 resin was extracted again with 30 mL of distilled acetone for 60 min and filtered through the same folded filter. The combined extracts were transferred into a 100 mL round bottom flask. Acetone was evaporated using a rotary evaporator and residual water was evaporated at 20 mbar until the residue in the flask was completely dry. The residue was taken up in 550 μL of methanol (Chromasolv HPLC grade, Sigma Aldrich, St. Louis, MO, USA) and transferred into a 1.5 mL Eppendorf tube. This tube was centrifuged with a table centrifuge (Hitachi, Tokyo, Japan) at 15,000 rpm for 2 min to remove residual insolubilities such as salts, cell debris, and XAD fragments. The residual extract was diluted 1:10 for UHPLC-hrMS analysis.

### 2.3. UPLC-hrMS Analysis

UPLC-coupled MS analysis was performed on a Dionex (Germering, Germany) Ultimate 3000 RSLC system using a Waters (Eschborn, Germany) BEH C18 column (50 × 2.1 mm, 1.7 µm) equipped with a Waters VanGuard BEH C18 1.7 µm guard column. Separation of 1 µL sample was achieved by a linear gradient from (A) H_2_O + 0.1% FA to (B) acetonitrile (ACN) + 0.1% formic acid (FA) at a flow rate of 600 µL/min and a column temperature of 45 °C. UV spectra were recorded by a DAD in the range from 200 to 600 nm. The LC flow was split to 75 µL/min before entering the maXis 4G hrToF mass spectrometer (Bruker Daltonics, Bremen, Germany). Additional device parameters and settings applied for LC separation, MS data acquisition, and processing are described in the SI.

### 2.4. Purification of Myxoquinones

Purification of myxoquinone 739 and 825 was carried out using a gradient LC system with a Waters Acquity CSH C18 250 × 10 mm 5 µm column with the eluents H_2_O + 0.1% FA as A and ACN + 0.1% FA as B, a flow rate of 5 mL/min, and the column kept at 30 °C. Further chromatographic details are described in the SI. Myxoquinone A and B were detected by UV absorption at 270 nm and purification was performed by time dependent fraction collection. After evaporation, the myxoquinones 739 and 825 were obtained from fraction as pale yellowish amorphous solids. Myxoquinones 843 and 861 were isolated by semi-preparative reverse phase chromatography with a similar setup but using a Phenomenex Jupiter Proteo 250 × 10 mm, 4 µm, 30 Å column; flow rate 6 mL/min. After evaporation, the myxoquinones 843 and 861 are obtained as pale yellowish amorphous solids. The purified compounds were dried and stored in air-tight glass vials at −20 °C.

### 2.5. NMR Measurements

Briefly, 1D and 2D NMR data used for structure elucidation of the myxoquinone derivatives were acquired in CDCl_3_ on a Bruker Ascend 700 spectrometer equipped with a TCI 5 mm probe head (^1^H at 700 MHz, ^13^C at 175 MHz) and Bruker Ultra Shield 500 spectrometer equipped with a TCI 5 mm probe head (^1^H at 500 MHz, ^13^C at 125 MHz). All observed chemical shift values (δ) are given in in ppm and coupling constant values (J) in Hz. Standard pulse programs were used for HMBC, HSQC, gCOSY, and DQF COSY experiments. HMBC experiments were optimized for 2,3 J_C-H_ = 6 Hz. The spectra were recorded in CDCl_3_ and chemical shifts in the solvent signals at δH 7.27 ppm and δC 77.0 ppm were used as reference signals for spectra calibration. The measurements were conducted in a 5 mm Shigemi tube (Shigemi Inc., Allison Park, PA, USA).

### 2.6. Genome Sequencing

The detailed protocol for genomic DNA isolation was included in the SI. The genome of strain MCy9049 was sequenced using Illumina sequencing technology. Strain MCy9003 was sequenced with a PacBio RS II device using a single SMRT cell. The raw sequence reads were assembled in the SMRT portal software as recommended by the manufacturer.

## 3. Results

### 3.1. Classification of the Producer Organisms

**Figure 2:**
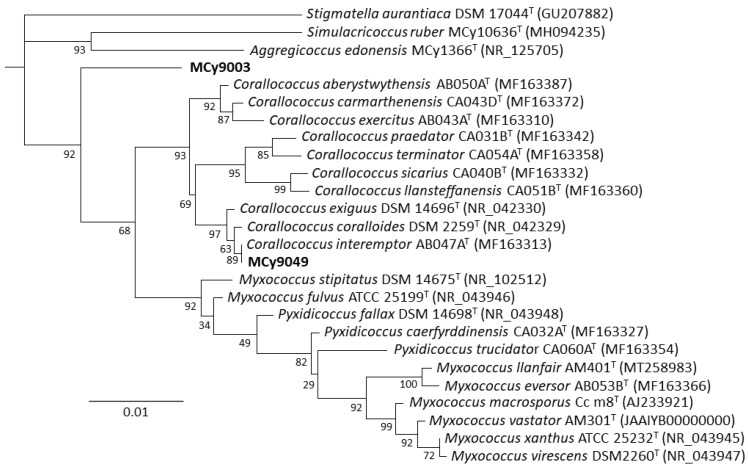
Phylogenetic tree (constructed using RAxML) inferred from 16S rRNA gene sequence showing the position of strains MCy9003 and MCy9049 in the *Myxococcaceae* clade. The sequence of *Stigmatella aurantiaca* DSM 17044^T^ was used as an outgroup to root the tree. GenBank accession numbers are given in parentheses. Bar, 0.01 nucleotide substitution per site.

**Figure 3:**
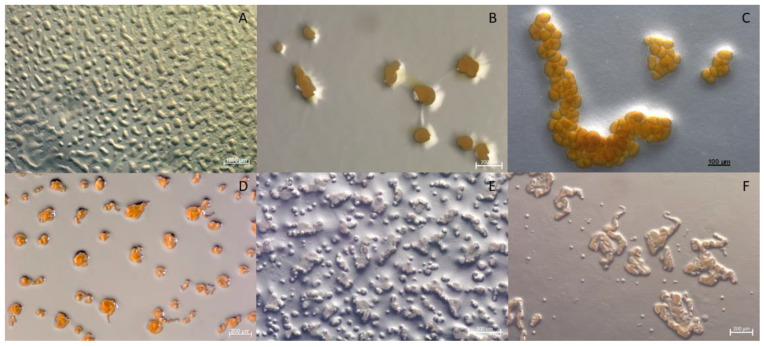
Comparison of fruiting body morphology observed from strains MCy9003 and MCy9049 to the related species in the family *Myxococcaceae*: (**A**) Strain MCy9003 fruiting body-like aggregates. (**B**) *Myxococcus macrosporus* DSM 14697^T^. (**C**) *Pyxidicoccus fallax* DSM 14698^T^. (**D**) Strain MCy9049. (**E**) *Corallococcus exiguus* DSM 14696^T^. (**F**) *C. coralloides* DSM 2259^T^. All strains were photographed after growth in VY/2 agar.

In this study, we reveal two myxobacterial strains as a source of the myxoquinones, a series of new vitamin K derived metabolites. The first myxobacterial strain, MCy9003, shows closest 16S rRNA gene similarity with *Myxococcus fulvus* ATCC 25199^T^ (98.20%), *Pyxidicoccus fallax* DSM 14698^T^ (98.13%), and *Myxococcus macrosporus* DSM 14697^T^ (97.78%). Phylogenetic analysis revealed its positioning in the phylogenetic tree separate from *Corallococcus*, *Myxococcus*, and *Pyxidicoccus* within the *Myxococcaceae* family and, thus, suggests it represents a novel taxon (Figure 2). Myxobacteria are known for their fruiting body phenotypes that can be used as an additional tool to aid taxonomic classification. Compared with the closest type strains, the fruiting body morphology of the novel strain is remarkably different supporting the uniqueness of this myxobacterial isolate (Figure 3A–C). The MCy9003 genome sequence was determined as a single contig representing the closed circular bacterial chromosome spanning over 11.2 million base pairs.

The second new strain, MCy9049, appears to be close to (99.86% similarity) *Corallococcus exiguus* DSM 14696^T^ and *C. coralloides* DSM 2259^T^ as analyzed by BLAST search using the 16S rRNA gene sequence. Based on phylogenetic analysis the strain clusters within the *Corallococcus* clade and is positioned branching with *C. interemptor* AB047A^T^. Phenotypic analysis of the fruiting bodies supports the strain’s placement within the *Corallococcus* clade as it is found to have fruiting bodies not enclosed in a sporangiole exhibiting horn-like projections (Figure 3D–F). Assembly of the sequence reads obtained from genomic DNA of MCy9049 resulted in a fragmented genome sequence consisting of 17 contigs with a total length of 9.87 million base pairs.

### 3.2. Identification, Isolation, and Bioactivity Evaluation of Four New Myxoquinones

*Corallococcus* sp. MCy9049 and *Myxococcaceae* sp. MCy9003 were analyzed for novel secondary metabolites. Fermentation of these strains combined with LC-MS analysis and subsequent feature-based PCA according to a previously established procedure revealed a large family of unknown compounds with similar MS² fragmentation patterns (Figures S4–S7) [13]. In addition, all putative family members exhibited strong and peculiar UV absorption spectra indicative of a conserved chromophore for both the myxoquinones 843 and 861 as well as the myxoquinones 739 and 825 (Figures S2 and S3). These observations sparked further interest in these compounds, which were thus set as targets for isolation and structure elucidation.

**Figure 4:**
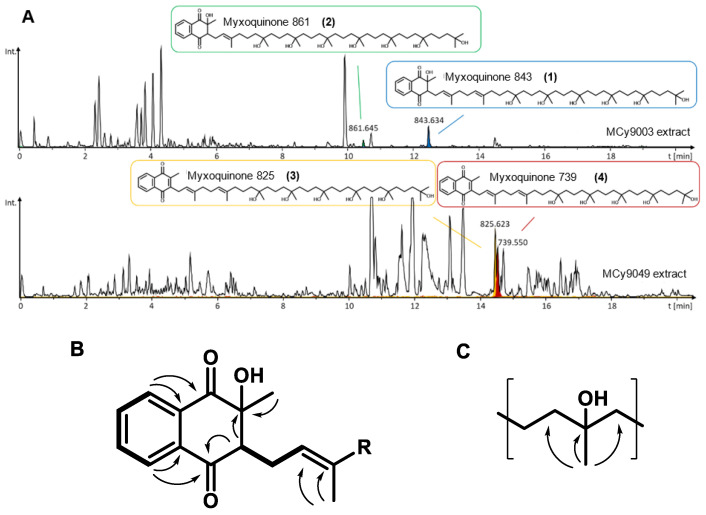
(**A**) LC-*hr*MS chromatograms showing production of the four myxoquinone derivatives by the respective myxobacterial strains. (**B**) Key COSY correlations (bold lines) and HMBC correlation (arrows) of the hydroxylated naphthoquinone system, and of the hydroxylated prenyl unit (**C**).

In order to perform full structure elucidation on the myxoquinones by NMR and to supply enough purified materials for bioactivity evaluation, *Corallococcus* sp. MCy9049 and *Myxococcaceae* sp. MCy9003 were cultivated in a larger scale batch fermentation in shake flasks (see SI). A series of compound purification steps consisting of liquid/liquid partitioning followed by semi-preparative HPLC with UV detection of the compounds led to the isolation of the four myxoquinone congeners named myxquinone 843, 861, 825, and 739 (Figure 4A), which were obtained as yellow solids. The purified myxoquinones were subjected to bioactivity testing against *Candida albicans*, *Pichia anomala*, *Citrobacter freundii*, *Acinetobacter baumanii*, *Staphyllococcus aureus*, *Bacillus subtilis*, *Escherichia coli*, *Pseudomonas aeruginosa*, and *Mycobacterium smegmatis* strains as well as HCT 166 and KB3.1 cell lines (Table S11). However, all four compounds lacked biological activity against any of the tested strains and cell lines at concentrations up to 64 µg/mL.

### 3.3. Full Structure Elucidation

For Myxoquinone 843 (**1**), the sum formula of C_51_H_86_O_9_ was deduced from *hr*MS. The proton spectrum showed resonances characteristic for aromatic, as well as olefinic protons between δ_H_ 5.09 and 8.12. In addition, signals between δ_H_ 1.19 and 3.06 indicated the presence of distinct methylene groups and methyl groups. Moreover, inspection of the HSQC spectrum revealed presence of overlapping methylene groups at (δ_C_ 42.0–43.0, δ_H_ 1.42–1.47) and (δ_C_ 18.0–19.0, δ_H_ 1.42–1.47) and methyl groups at (δ_C_ 26.5–27.4, δ_H_ 1.15–1.24), which indicates a repetitive moiety to be present in the molecule. Full spectral details are given in the SI (Tables S7–S10, Figures S8–S24), and key NMR correlations are highlighted in Figure 4B,C. By analysis of HMBC and COSY spectra, we could identify the aromatic proton resonances derived from a 2-methyl-naphtoquinone ring that is hydroxylated at position two. Similar to other quinone type molecules a prenyl sidechain is linked to position 3 of the ring. The chain length was determined by the *hr*MS-derived sum formula to consist of eight prenyl units. In contrast to other quinone type molecules, the last six prenyl units are hydroxylated, which is indicated by HMBC correlations from the prenyl methyl groups to a quaternary carbon with characteristic shift values between δ_C_ 72 and 73. The two prenyl units next to the naphthoquinone ring were not hydroxylated as seen by characteristic olefinic protons resonances of δ_H2’_ 5.35 and δ_H6’_ 5.09.

The other isolated myxoquinone derivatives differ in hydroxalation state and chain length: In myxoquinone 861 (**2**), the last seven prenyl units are hydroxlyated as seen by an additional hydroxyl group in the sum formula of C_51_H_88_O_10_ and only one olefinic proton signal at δ_H__2’_ 5.35. In contrast myxoquinone 825 (**3**), the last six prenyl groups are hydroxylated as in **1**, but here the naphthoquinone ring is not hydroxylated, which is indicated by the sum formula of C_51_H_84_O_8_ and overlapping aromatic proton signals at δ_H7,6_ 7.70 and δ_H5,8_ 7.70, while proton signal of ring position **3,** as in **1** and **2,** is missing. Myxoquinone 739 (**4**) is structurally similar to **3**, but contains seven instead of eight prenyl units as indicated by the sum formula of C_46_H_74_O_7_.

### 3.4. Distribution of the Myxoquinones among Myxobacteria

Many known myxobacterial secondary metabolites have been shown to follow a species- or genus-specific production pattern, and thus we sought to determine how widespread the new myxoquinones occur among myxobacteria [14]. In addition to the mentioned *Corallococcus* sp. MCy9049 and *Myxococcaceae* sp. MCy9003, we observed production of all four myxoquinone congeners also in the myxobacterial model strain *M. xanthus* DK1622. We therefore searched our in our in-house secondary metabolome database containing approximately 2600 LC-MS datasets from diverse myxobacteria in order to catalog the occurrence of myxoquinones based on retention time, exact mass and isotope pattern matching [14]. Search parameters were retention time deviation below 0.3 min and exact mass deviation below 10 ppm (see SI). This search revealed that myxoquinones occur only in *Cystobacterineae* and not in other myxobacterial taxa, while LC-MS analysis shows them to occur in most of the *Cystobacterineae* screened. On the family level, the myxoquinones seem to be most prevalent among the *Myxococcaceae* with myxoquinone 825 occurring abundantly in *M. xanthus* and *C. coralloides* strains, myxoquinone 739 occurring mostly in *M. fulvus* and *C. coralloides* strains, while myxoquinone 843 and 861 occur frequently in *M. stipitatus* strains. To underpin these findings by a complementary genomic survey, we analyzed the distribution of the myxoquinone biosynthetic gene cluster among myxobacteria by BLAST search and pairwise alignments (see Figure S1). We found that the conserved myxoquinone gene cassette likely responsible for myxoquinone biosynthesis occurs widespread among *Cystobacterineae*. The prevalence of the *mxq* BGC is summarized in Table S6.

## 4. Discussion

Myxobacteria remain underexplored prokaryotes and there seems to be greater than anticipated uncultured phylogenetic diversity based on metagenomic analyses [15]. Over the last decade, new genera and even families were discovered and brought to culture, which expands the previous knowledge of myxobacterial taxonomy, ecology, biology, and physiology. *Sorangium* [16], *Corallococcus* [17], *Myxococcus*, and *Pyxidicoccus* [18] are named here as examples for expanded diversity after several new species were described in the last years. The last three genera belong to the *Myxococcaceae*, a myxobacterial family that appears still underestimated in terms of species richness and associated metabolite abundance, a notion that is also underpinned by the present study.

### 4.1. Biosynthetic Origin of the Myxoquinones and Biosynthesis Proposal

Due to their unusual oxygenated scaffolds, the new myxoquinones from *Myxococcaceae* reported here differ significantly from phylloquinones and menaquinones. Still, the myxoquinone core scaffold shows significant similarity to the vitamin K scaffold, indicating a potential common biosynthetic intermediate. As biosynthetic genes for menaquinone biosynthesis are clustered in bacterial model organisms such as, e.g., *B. subtilis*, we searched the genomes of the producer organisms *Corallococcus* sp. MCy9049 and *Myxococcaceae* sp. MCy9003 and were able to identify a locus containing a cluster of biosynthesis genes similar to the menaquinone pathway (Table S5) [19].

**Figure 5:**
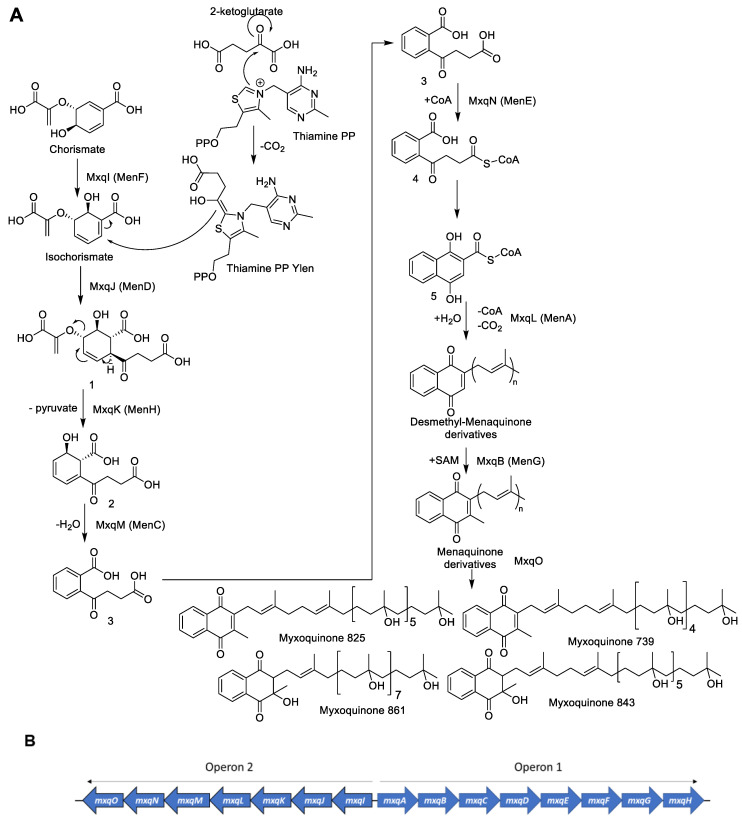
(**A**) Proposed biosynthesis of the myxoquinones. (**B**) Schematic overview for the identified myxoquinone biosynthetic gene cluster from MCy9049.

**Scheme 1:**
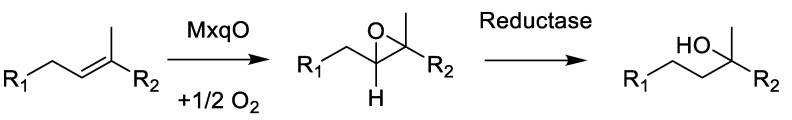
Formal hydration reaction by epoxidation and subsequent epoxide reduction to create the myxoquinones.

Based on our findings, we propose the biosynthetic pathway for myxoquinones as shown in Figure 5A. The biosynthesis is initiated with the formation of the 1,4-naphthoquinone core structure as has been described for menaquinone biosynthesis [20]. To facilitate the comparison between myxoquinone and menaquinone biosynthesis we annotated the Mxq Proteins that have analogues performing the same biosynthetic steps during menaquinone biosynthesis with the corresponding Men genes (see Supplementary Materials). Biosynthesis starts by MxqI, a MenF type chorismate isomerase enzyme that transforms chorismate from primary metabolism into isochorismate [21]. Isochorismate is subsequently elongated by a succinyl group via a nucleophilic addition of a ketoglutarate moiety catalyzed by the thiamine dependent MenD analogue MxqJ. The carbonyl carbon is switched in its polarity by decarboxylative coupling to Vitamin B2, which forms a thiamine ylen/ylide structure, able to carry out nucleophilic substitutions (Figure 5) [22]. The MenH analogue MxqK subsequently eliminates pyruvate from the substituted isochorismate of intermediate 1 in an elimination reaction to form intermediate 2. This intermediate is subsequently aromatized in a dehydration reaction catalyzed by MxqM, a MenC type aromatase [23]. The produced 1,2 substituted aromatized intermediate 3 is subsequently transformed into the CoA-ester 4 by the MenE type CoA ligase MxqN that can subsequently cyclize by intramolecular Claisen condensation to form the 1,4-naphthoquinol core structure 5 that undergoes CoA-ester hydrolysis, followed by decarboxylation [20,24]. This intermediate can be oxidized to the 1,4-naphtoquinone afterwards. A UbiA/MenA type prenyl transferase transforms the naphthoquinone core into demethyl-menaquinone derivatives of different side chain lengths consisting of seven to nine isoprene units (Figure 5) [25]. This step is one of the crucial biosynthetic steps to afford the structural diversity observed in the isolated compounds. In the myxoquinone case, this naphthoquinone prenyl transferase is encoded on *mxqL*. These intermediates are then transformed into the corresponding menaquinone derivative by a C3 methyl transfer carried out by the MenG type SAM-dependent methyl transferase MxqB [26]. The next step in myxoquinone biosynthesis is the formal hydration of the isoprene units to the tertiary alcohols observed in the myxoquinones as indicated in Scheme 1, which has no precedent in other naphthoquinone biosyntheses.

This reaction is likely to occur as a two-step mechanism since direct hydroxylation of double bonds requires a strong oxidative redox-potential. We reason that this reaction is conducted by the concerted effort of an epoxidizing enzyme such as cytochrome P450 followed by a reductive epoxide ring opening, as this could explain the formation of the tertiary alcohols; albeit, currently, we cannot provide experimental proof for this hypothesis. A plausible candidate enzyme for this reaction would be MxqO, a Cytochrome P450 enzyme that can form OH groups in double epoxidation reactions by forming the thermodynamic Markovnikov products observed in the myxoquinones after reduction. Since we could not identify any NAD(P)H reductases in the *mxq* cluster, which could be expected to perform this reaction, the reductive part of the reaction cascade depicted in Scheme 1 remains elusive. We sought to support our considerations by targeted gene inactivation, but despite significant efforts we were unable to inactivate the mxp BGC by single crossover mutagenesis. Therefore, we conclude that the naphthoquinones produced by this BGC are either obligatory for the survival of these strains; albeit, we cannot rule out the alternative explanation that these new isolates are not amenable to genetic manipulation by established protocols, as myxobacterial strains are notoriously difficult to genetically manipulate.

### 4.2. On the Possible Biological Role of Myxoquinones

All myxobacterial genomes with detected *mxq* BGCs, of which the published strains in Table S6 are an excerpt, belonged to the *Cystobacterineae* suborder of myxobacteria. The widespread occurrence of myxoquinones within this group raises the question whether these molecules should be considered as secondary (or “specialized”) metabolites, or the compound family should rather be recognized as part of the primary metabolism in the host bacterium. The latter notion is supported by the finding that contrary to myxobacterial respiratory chain inhibitors that interfere with the electron transport chain in bacteria, such as the aurachins, stigmatellins [27], myxothiazols [28], or melithiazols [29] myxoquinones, neither showed cytotoxicity nor antibacterial activity against the tested pathogen model strains and eukaryotic cell lines. Therefore, it appears unlikely that the myxoquinones are defensive secondary metabolites the myxobacterium could utilize to fend off competing microorganisms. The bioactivity results instead point towards the hypothesis that these metabolites have evolved among *Cystobacterineae* to perform their tasks as redox co-factors and not as electron transport inhibitors. In fact, in our LC-MS data, we could not detect the simultaneous presence of menaquinones in myxobacterial extracts. Therefore, the possibility has to be considered that the menaquinone-like function in *Cystobacterineae* is entirely assumed by the myxoquinones.

The description of the new menaquinone derivatives highlights our, to date still limited, knowledge concerning the role of Vitamin K in the producer organisms. While the role of Vitamin K in the blood clotting cascade has been thoroughly characterized, its role in both bacteria and the chloroplast thylakoid are not yet fully understood [30]. The rather widespread appearance of the myxoquinones in the myxobacterial family *Myxococcaceae* combined with the complete lack of biological activity even at high concentrations against both human cancer cell lines as well as indicator microorganisms for common pathogens points towards their putative function as metabolites used for the upkeep of cell functions. Since the Vitamin K scaffold contains the naphthoquinone ring as a structure-determining feature that can readily exist in two distinct redox states, namely the quinone and the quinol forms, we reason that these metabolites likely play a vital role in redox processes in bacterial cells. Indeed, the better characterized natural quinones involved in redox processes such as ubiquinone or coenzyme Q10 are involved in a variety of redox functions, most notably the transport of protons and electrons during oxidative phosphorylation [31]. Contrary to eukaryotes, which are very much uniquely reliant on ubiquinone to fulfill the task of electron and proton transfer, bacteria seem to have evolved a much broader variety of quinone carriers [32]. *E. coli*, for example, uses both ubiquinone as well as menaquinone and demethyl-menaquinone as electron and proton carrier involved in various enzymatic redox reactions [33]. Quite the opposite idea has been drawn into focus in recent years: As Vitamin K congeners including myxoquinones seem to have no antimicrobial properties, but the respective pathways are essential for many microorganisms, their pathway constituting enzymes are increasingly seen as a target for anti-infectives themselves [34]. Such strategies may indeed become fruitful in the future as the remarkable conservation of the vitamin K pathway enzymes is also shown in this work.

## 5. Conclusions

In this work, we describe the myxoquinones as novel variants of Vitamin K occurring in the myxobacterial taxon *Cystobacterineae*, and in particular within strains of the *Myxococcus* and *Corallococcus* genera. These new Vitamin K derivatives are interesting from a structural point of view due to their extensive modification leading to multi-hydroxylation, which makes them considerably more water soluble when compared to phylloquinone or menaquinone. The new Vitamin K congeners are therefore a valuable addition to the structural diversity presented by the Vitamin K family [35]. Myxoquinones may thus open a new avenue of application in medical use, if they can function as viable replacements of menaquinone or phylloquinone in the human body to start the blood clotting cascade. While it seems likely that the myxoquinones 825 and 739 should be able to be epoxidized and, thus, start the blood clotting cascade; for myxoquinones 843 and 861 a re-aromatization of the naphthoquinone system would need to occur. If any or both of the myxoquinone types were able to replace vitamin K in humans lacking appropriate vitamin levels, and in addition be tolerable in respective clinical settings, these compounds could represent a potential treatment option as their highly oxygenated tail structure might facilitate improved vitamin K uptake.

## Data Availability

The sequence information for biosynthetic gene clusters from strains MCy9003 and MCy9049 was deposited to GenBank.

## Supplementary Materials

The following are available online at https://www.mdpi.com/article/10.3390/microorganisms10030534/s1: growth media recipes (Tables S1–S4), an in-detail description of all utilized analytical chemistry methods including HPLC and uHPLC-MS methods, molecular biology and bioactivity testing protocols, in silico analyses on gene and protein levels (Tables S5, S6 and S11; Figure S1), as well as all relevant spectral data for structure elucidation (Figures S2–S24; Tables S7–S10).
