# Supplementary material for: Myxobacteria of the Cystobacterineae Suborder Are Producers of New Vitamin K2 Derived Myxoquinones
Source: Microorganisms. 2022 Feb 28;10(3):534. doi: 10.3390/microorganisms10030534 (PMC8955186; doi:10.3390/microorganisms10030534)
Supplement: Supplementary file 1 [file microorganisms-10-00534-s001.zip › microorganisms-1566209 suppl final.pdf]

Myxobacteria of the *Cystobacterinae* Suborder are Producers of New Vitamin K_2 Derived Myxoquinones

Fabian Panter[a,b,c], Alexander Popoff[a,b], Ronald Garcia[a,b], Daniel Krug[a,b] and Rolf Müller* [a,b,c]

[a] Department of Microbial Natural Products, Helmholtz-Institute for Pharmaceutical Research Saarland (HIPS), Helmholtz Centre for Infection Research (HZI) and Department of Pharmacy, Saarland University, Campus E8 1, 66123 Saarbrücken, Germany

[b] German Centre for Infection Research (DZIF), Partner Site Hannover - Braunschweig, Germany

[c] Helmholtz International Lab for Anti-Infectives, Campus E8 1, 66123 Saarbrücken, Germany

Supporting Information

Table of Contents

1 MYXOBACTERIAL GROWTH CONDITIONS ... 3

1.1 MYXOBACTERIAL CULTURE MEDIA ... 3

1.2 MYXOBACTERIAL FERMENTATION CONDITIONS FOR LC-MS ANALYSIS ... 4

2 ANALYTICAL METHODS USED IN THIS WORK ... 5

2.1 METABOLITE EXTRACTION PROCEDURE FOR ANALYTICAL SCALE EXTRACTIONS ... 5

2.2 STANDARDIZED UHPLC MS CONDITIONS ... 5

2.3 METHODOLOGY FOR STATISTICS-BASED METABOLOME FILTERING ... 6

2.4 ACQUISITION PARAMETERS FOR ACQUIRING HIGH-RESOLUTION TANDEM MS DATA ... 6

2.5 MYXOQUINONES DISTRIBUTION AMONG MYXOBACTERIAL LC-MS DATA ... 6

3 *CORALLOCOCCUS SP.* MCY9049 AND *MYXOCOCCACEAE SP.* MCY9003 ... 12

3.1 PREPARATION OF GENOMIC DNA FOR GENOME SEQUENCING ... 13

3.2 RESULTS OF GENOME SEQUENCING OF MCY9049 AND MCY9003 WILD TYPE ... 14

3.2.1 Sequencing results of Corallococcus sp. MCy9049 ... 14

3.2.2 Sequencing results of Myxococcaceae sp. MCy9003 ... 14

3.3 *IN-SILICO* BLAST ANALYSIS OF THE MYXOQUINONE BIOSYNTHETIC GENE CLUSTER ... 15

4 ISOLATION AND STRUCTURE ELUCIDATION OF THE MYXOQUINONES ... 15

4.1 FERMENTATION, ISOLATION AND PURIFICATION OF THE MYXOQUINONES ... 17

4.1.1 Cultivation and extraction of Myxoquinone 739 and 825 from MCy9049 ... 17

4.1.2 Purification of Myxoquinone 739 and 825 ... 17

4.1.3 Cultivation and extraction of Myxoquinone 843 and 861 from MCy9003 ... 18

4.2 UV SPECTRA OF THE MYXOQUINONES ... 18

4.3 TANDEM MS SPECTRA OF THE MYXOQUINONES ... 19

4.4 NMR BASED STRUCTURE ELUCIDATION ... 20

4.4.1 NMR conditions and spectroscopic data ... 20

4.4.2 Structure elucidation of the Myxoquinone derivatives ... 21

4.4.3 Tabulated NMR signals for every myxoquinone derivative ... 23

5 NMR SPECTRA EMPLOYED IN MYXOQUINONE STRUCTURE ELUCIDATION ... 31

6 BIOLOGICAL ASSAY CONDITIONS ... 47

7 REFERENCES ... 48

1 Myxobacterial growth conditions

1.1 Myxobacterial culture media

Table S1. *Recipe for VY/2 medium*

VY/2 – Medium			
Amount	Ingredient	Concentration	Supplier
5 g/L	Yeast (entire cells)	-	Wonnemeyer
1 g/L	$CaCl_2$	-	Sigma Aldrich
1 g/L	$MgSO_4 \cdot 7H_2O$	-	Grüssing
10 mL/L	TRIS • HCl pH 8	1M	Sigma Aldrich
100 µL/L	Sterile Vit. B12 solution (added after autoclaving)	1 mg/mL	Roth
200 µL/L	Sterile Fe-EDTA solution (added after autoclaving)	8 mg/mL	Sigma Aldrich
Dissolved in milli-Q. Water, pH adjusted to 7.2 with 1N KOH			

Table S2. *Recipe for VY medium*

VY – Medium			
Amount	Ingredient	Concentration	Supplier
10 g/L	Yeast (entire cells)	-	Wonnemeyer
5 g/L	Soluble Starch	-	Roth
1 g/L	$CaCl_2$	-	Sigma Aldrich
1 g/L	$MgSO_4 \cdot 7H_2O$	-	Grüssing
10 mL/L	TRIS • HCl pH 8	1M	Sigma Aldrich
100 µL/L	Sterile Vit. B12 solution (added after autoclaving)	1 mg/mL	Roth
200 µL/L	Sterile Fe-EDTA solution (added after autoclaving)	8 mg/mL	Sigma Aldrich
Dissolved in milli-Q. Water, pH adjusted to 7.2 with 1N KOH			

Table S3. *Recipe for PYGS medium*

PYGS – Medium			
Amount	Ingredient	Concentration	Supplier
20 g/L	Yeast (entire cells)	-	Wonnemeyer
10 g/L	Potato Starch	-	Sigma Aldrich
1 g/L	$CaCl_2$	-	Sigma Aldrich
1 g/L	$MgSO_4 \cdot 7H_2O$	-	Grüssing
10 mL/L	TRIS • HCl pH 8	1M	Sigma Aldrich
100 µL/L	Sterile Vit. B12 solution (added after autoclaving)	1 mg/mL	Roth
200 µL/L	Sterile Fe-EDTA solution (added after autoclaving)	8 mg/mL	Sigma Aldrich
Dissolved in milli-Q. Water, pH adjusted to 7.6 with 1N KOH			

Table S4. *Recipe for CyHv3 medium*

CyHv3 media			
Amount	Ingredient	Concentration	Supplier
2 g/L	Soytone	-	BD
3 g/L	Casitone	-	Sigma Aldrich
1 g/L	$CaCl_2$ $2H_2O$	-	Sigma Aldrich
2 g/L	Glucose		Sigma Aldrich
8 g/L	Starch		Roth
1.5 g/L	Yeast extract		BD
1 g/L	$MgSO_4 \bullet 7H_2O$	-	Grüssing
10 mL/L	HEPES	1M	Roth
200 µL/L	Sterile Fe-EDTA solution (added after autoclaving)	8 mg/mL	Sigma Aldrich
Dissolved in milli-Q. Water, pH adjusted to 7.6 with 1N KOH			

Myxobacteia cultivations were performed according to previously described procedures [1]. The myxobacterial strains were kept in agar culture both for storage over short amounts of time. The agar media used are VY/2 agar, which is prepared by adding 14 g/L agarose (BD) to VY/2 medium preparations before autoclaving.

1.2 Myxobacterial fermentation conditions for LC-MS analysis

Cultures for UHPLC-*hr*MS analysis are grown in 300 mL shake flasks containing 50 mL of VY/2, VY, PYGS or CyHv3 medium for both *Corallococcus sp.* MCy9049 and *Myxococcaceae sp.* MCy9003 inoculated with 1 mL of pre culture. After inoculation the medium is supplemented with 2% of sterile XAD-16 adsorber resin (Sigma Aldrich) suspension in water to bind secondary metabolites in the culture medium and limit auto toxicity. Small scale cultures were grown for 10-12 days until the fermentation medium has completely cleared up except for the myxobacterial biofilm clumps and XAD-16 resin particles. After fermentation the culture is pelleted in a 50 mL falcon at 6000 rcf for 10 minutes using an Eppendorf falcon table centrifuge and stored at -20 °C until further use.

2 Analytical methods used in this work

2.1 Metabolite extraction procedure for analytical scale extractions

The frozen cell pellet is transferred into a 100 mL Erlenmeyer flask and a magnetic stirrer is added. 50 mL of acetone (fluka analytical grade, redistilled in house) are added onto the pellet and the mixture is stirred for 60 min on a magnetic stirrer. The acetone extract is left to settle in order to sediment cell debris and XAD resin for a second extraction step. The supernatant is filtered with a 125-micron folded filter keeping cell pellet and XAD-16 resin in the Erlenmeyer flask for a second extraction step. The residual pellet and XAD-16 resin is extracted again with 30 mL of distilled acetone for 60 min on a magnetic stirrer and filtered through the same folded filter. The combined extracts are transferred into a 100 mL round bottom flask. The acetone is evaporated using a rotary evaporator at 260 mbar and 40 °C water bath temperature. The residual water is evaporated at 20 mbar until the residue in the flask is completely dry. The residue is taken up in 550 µL of methanol (Chromasolv HPLC grade, Sigma Aldrich) and transferred into an 1.5 mL Eppendorf tube. This tube is centrifuged with a Hitachi table centrifuge at 15000 rpm for 2 minutes to remove residual insolubilities such as salts, cell debris and XAD fragments. The residual extract is diluted 1:10 for UHPLC-*hr*MS analysis.

2.2 Standardized UHPLC MS conditions

UPLC-*hr*MS analysis is performed on a Dionex (Germering, Germany) Ultimate 3000 RSLC system using a Waters (Eschborn, Germany) BEH C18 column (50 x 2.1 mm, 1.7 µm) equipped with a Waters VanGuard BEH C18 1.7 µm guard column. Separation of 1 µL sample is achieved by a linear gradient from (A) H_2O + 0.1 % FA to (B) ACN + 0.1 % FA at a flow rate of 600 µL/min and a column temperature of 45 °C. Gradient conditions are as follows: 0 – 0.5 min, 5% B; 0.5 – 18.5 min, 5 – 95% B; 18.5 – 20.5 min, 95% B; 20.5 – 21 min, 95 – 5% B; 21-22.5 min, 5% B. UV spectra are recorded by a DAD in the range from 200 to 600 nm. The LC flow is split to 75 µL/min before entering the Bruker Daltonics maXis 4G *hr*ToF mass spectrometer (Bremen, Germany) equipped with an Apollo II ESI source. Mass spectra are acquired in centroid mode ranging from 150 – 2500 m/z at a 2 Hz full scan rate. Mass spectrometry source parameters are set to 500 V as end plate offset; 4000 V as capillary voltage; nebulizer gas pressure 1 bar; dry gas flow of 5 l/min and a dry temperature of 200 °C. Ion transfer and quadrupole settings are set to funnel RF 350 Vpp.; multipole RF 400 Vpp as transfer settings and ion energy of 5 eV as

well as a low mass cut of 300 m/z. Collision cell is set to 5.0 eV and pre-pulse storage time is set to 5 µs. Spectra acquisition rate is set to 2 Hz. Calibration is done automatically before every LC-MS run by injection of sodium formate and calibration on the respective clusters formed in the ESI source. All MS analyses are acquired in the presence of the lock masses ($C_{12}H_{19}F_{12}N_3O_6P_3$, $C_{18}H_{19}F_{24}N_3O_6P_3$ and $C_{24}H_{19}F_{36}N_3O_6P_3$) which generate the $[M+H]^+$ ions of 622.0289; 922.0098 and 1221.9906.

2.3 Methodology for statistics-based metabolome filtering

In order to detect all secondary metabolites stemming from the wild-type organisms MCy9049 and MCy9003, we analyzed extracts and the corresponding media blanks in an unbiased principal component analysis (PCA) adapted from Krug *et al.* and Panter *et al.* [2,3]. For this purpose, LC-MS chromatograms of 3 independently generated extracts of both the strain and the medium blank are measured as 2 technical replicates each, giving a total number of 6 LC-*hr*MS chromatograms per strain. To obtain all molecular features in the 6 LC-*hr*MS chromatograms of the bacterial extracts and the 6 LC-*hr*MS chromatograms of the corresponding medium blank extracts, the T-ReX-3D molecular feature finder implemented in Bruker Metaboscape 4.01 was used. Compound detection parameters intensity threshold is set to 10000, mz threshold to 0.005 Da and minimum compound length to 4 spectra. PCA t-test tables are created with the built in PCA t-test routine and filtered according to 6 appearances in the extract chromatograms and 0 appearances in the medium blank extract chromatograms.

2.4 Acquisition parameters for acquiring high-resolution tandem MS data

LC and MS conditions for SPL guided MS/MS data acquisitions are kept constant according to section standardized UHPLC-MS conditions. MS/MS data acquisition parameters are set to exclusively fragment scheduled precursor list entries. SPL tolerance parameters for precursor ion selection are set to 0.2 minutes and 0.05 m/z in the SPL MS/MS method. The method picks up to 2 precursors per cycle, applies smart exclusion after 5 spectra and performs CID and MS/MS spectra acquisition time ramping. CID Energy is ramped from 35 eV for 500 m/z to 45 eV for 1000 m/z and 60 eV for 2000 m/z. MS full scan acquisition rate is set to 2 Hz and MS/MS spectra acquisition rates are ramped from 1 to 4 Hz for precursor ion intensities of 10 kcts. to 1000 kcts.

2.5 Myxoquinones distribution among myxobacterial LC-MS data

We searched our in our in-house secondary metabolome database containing approximately 2600 LC-MS datasets from diverse myxobacteria in order to catalog the occurrence of myxoquinones based on retention time, exact mass and isotope pattern matching, using the approach and technology as previously described in Hoffmann *et al.* [4]. Search parameters were retention time deviation below 0.3 min. and exact mass deviation below 10 ppm. Search results are shown below exemplified by the first 100 hits for Myxoquinones 825 and 843 respectively, sorted by chromatographic peak areas (in descending order).

Query 1: Myxoquinone 825

Meta_ID	Strain	Iontype	Δ ppm	ΔmDa	Rt.diff	Peak Area
META2894	MCy10674	[M+H]+	2.59	2.14	0.07	19154616
META2855	MCy10703	[M+H]+	2.07	1.71	0.02	9370891
META2867	MCy9543	[M+H]+	2.5	2.06	0.01	8268983
META2854	MCy10700	[M+H]+	2.18	1.8	0.01	6075233
META2898	MCy10743	[M+H]+	2.31	1.91	0.06	5460484
META2868	MCy9545	[M+H]+	1.98	1.63	0	4518859
META2862	MCy9534	[M+H]+	1.77	1.46	0.02	4297992
META2864	MCy9537	[M+H]+	2.07	1.71	0.01	4065526
META2823	MCy9066	[M+H]+	1	0.83	0.05	3516224
META925	MCy9541	[M+H]+	1.31	1.08	0.03	3427459
META924	MCy9540	[M+H]+	2.03	1.67	0.01	3174831
META2883	MCy9178	[M+H]+	2.45	2.02	0.02	2431815
META2861	MCy9482	[M+H]+	2.07	1.71	0.01	2154062
META907	MCy10979	[M+H]+	1.74	1.44	0.04	2130931
META3625	MCy11770	[M+H]+	0.66	0.54	0.07	1977287
META2811	MCy10704	[M+H]+	1.23	1.02	0.05	1922914
META2825	MCy9068	[M+H]+	2.12	1.75	0.04	1762379
META2932	MCy9201	[M+H]+	1.42	1.17	0.09	1753132
META3410	MCy11646	[M+H]+	1.13	0.94	0.09	1692509
META3773	MCy11951	[M+H]+	0.49	0.41	0	1635720
META3774	MCy11952	[M+H]+	0.43	0.35	0	1588033
META3411	MCy11647	[M+H]+	0.97	0.8	0.1	1536720
META2826	MCy9070	[M+H]+	1.83	1.51	0.05	1512895
META3384	MCy11382	[M+H]+	0.77	0.63	0.11	1501385
META3598	MCy11666	[M+H]+	0.88	0.72	0.05	1430358
META903	MCy10953	[M+H]+	2.77	2.28	0.03	1426465
META1852	Mxx5	[M+H]+	3.04	2.51	0.09	1360218
META3801	MCy11957	[M+H]+	0.47	0.38	0	1336770
META2899	MCy10933	[M+H]+	1.91	1.58	0.06	1314208
META3629	MCy11770	[M+H]+	0.43	0.35	0.2	1310950

META2827	MCy9071	[M+H]+	3.16	2.61	0.04	1302329
META3366	MCy11653	[M+H]+	1.15	0.95	0.1	1248761
META3775	MCy11953	[M+H]+	0.57	0.47	0.01	1239908
META3608	MCy11742	[M+H]+	0.83	0.68	0.05	1184679
META3654	MCy11812	[M+H]+	0.75	0.62	0.03	1148907
META3664	MCy11812	[M+H]+	0.75	0.62	0.03	1148907
META3632	MCy11561	[M+H]+	0.37	0.31	0.03	1110500
META3401	MCy11586	[M+H]+	0.5	0.41	0.11	1101711
META3367	MCy11654	[M+H]+	1.12	0.92	0.09	1099634
META3284	Mxf26	[M+H]+	1.27	1.05	0.11	1071274
META902	MCy10952	[M+H]+	1.89	1.56	0.04	1045776
META3009	MCy9003	[M+H]+	1.42	1.18	0	1038439
META3548	MCy10732	[M+H]+	0.33	0.27	0.05	1017004
META2812	MCy10728	[M+H]+	2.53	2.09	0.04	1013821
META2910	MCy11166	[M+H]+	2.46	2.03	0.08	946782
META3385	MCy11382	[M+H]+	0.31	0.25	0.1	946605
META3553	MCy11720	[M+H]+	0.47	0.39	0.04	928569
META3621	MSr11624	[M+H]+	4.5	3.72	5.87	889071
META3605	MCy11666	[M+H]+	1.36	1.12	0.05	860779
META3653	MCy11812	[M+H]+	0.99	0.82	0.04	858343
META3663	MCy11812	[M+H]+	0.99	0.82	0.04	858343
META3387	MCy11382	[M+H]+	1.3	1.08	0.1	844751
META3399	MCy11584	[M+H]+	0.52	0.43	0.11	844415
META3667	MCy9003	[M+H]+	0.83	0.68	0.17	796426
META3657	MCy9003	[M+H]+	0.83	0.68	0.17	796426
META3534	MCy10644	[M+H]+	0.91	0.75	0.04	789297
META3533	MCy10644	[M+H]+	0.43	0.36	0.05	772675
META3464	MCy11558	[M+H]+	1.42	1.17	0.04	743547
META3704	MCy10639	[M+H]+	0.79	0.66	0.08	728505
META3502	MCy11668	[M+H]+	0.41	0.34	0.07	716621
META3379	MCy11382	[M+H]+	0.47	0.39	0.22	707472
META1688	Mxf253	[M+H]+	2.95	2.44	0.06	696110
META2839	MCy9097	[M+H]+	2.45	2.02	0.04	680771
META3798	MCy11957	[M+H]+	0.73	0.6	0.15	662663
META332	Mxf401	[M+H]+	1	0.82	0.07	658524
META3549	MCy10732	[M+H]+	0.97	0.8	0.05	609663
META2819	MCy9061	[M+H]+	2.32	1.92	0.03	592702
META3386	MCy11382	[M+H]+	0.93	0.77	0.26	592025
META3381	MCy11382	[M+H]+	1.29	1.07	0.1	583529
META904	MCy10954	[M+H]+	0.58	0.48	0.19	581917
META3516	MCy11308	[M+H]+	0.9	0.74	0.04	577075
META3627	MCy11770	[M+H]+	0.09	0.08	0.29	560104
META3794	MCy11957	[M+H]+	1.76	1.45	0.02	553456
META3607	MCy11666	[M+H]+	0.63	0.52	0.05	547000

META2971	MCy11281	[M+H]+	2.72	2.25	0.03	526403
META3718	MCy11909	[M+H]+	0.89	0.73	0.07	520823
META3651	MCy11812	[M+H]+	1.18	0.97	0.04	519885
META3661	MCy11812	[M+H]+	1.18	0.97	0.04	519885
META1153	Cce112	[M+H]+	1.52	1.25	0.01	501288
META3501	MCy11668	[M+H]+	1.06	0.88	0.06	492917
META248	Mxf39	[M+H]+	2.62	2.16	0.06	487974
META2145	Ccc578	[M+H]+	3.7	3.06	0.09	484748
META3005	MCy10649	[M+H]+	3.27	2.7	0.03	481502
META2499	Mxf173	[M+H]+	2.67	2.2	0.06	480385
META848	Mxf20	[M+H]+	1.79	1.48	0.01	479768
META2859	MCy9055	[M+H]+	3.55	2.93	0	476174
META3540	MCy10732	[M+H]+	0.49	0.4	0.04	464106
META1452	Mxf129	[M+H]+	1.7	1.41	0.05	460783
META918	MCy10727	[M+H]+	2.56	2.11	0.03	458993
META3551	MCy11720	[M+H]+	1.15	0.95	0.05	457640
META3368	MCy11661	[M+H]+	1.17	0.97	0.32	444897
META1060	Ccc869	[M+H]+	3.86	3.19	0.01	442523
META3537	MCy10644	[M+H]+	0.81	0.67	0.05	433551
META3531	MCy10644	[M+H]+	0.31	0.25	0.04	432286
META3792	MCy11957	[M+H]+	0.81	0.67	0.23	432058
META3535	MCy10644	[M+H]+	0.49	0.4	0.04	431040
META1685	Mxf9	[M+H]+	2.46	2.03	0.06	426726
META3515	MCy11305	[M+H]+	0.55	0.46	0.05	425781
META3050	MCy9487	[M+H]+	3.46	2.85	0.01	415054

Query 2: Myxoquinone 843

Meta_ID	Strain	Iontype	Δ ppm	ΔmDa	Rt.diff	Peak Area
META3629	MCy11770	[M+H]+	1.97	1.66	0.01	4091378
META3667	MCy9003	[M+H]+	1.78	1.5	0.02	2466654
META3657	MCy9003	[M+H]+	1.78	1.5	0.02	2466654
META2867	MCy9543	[M+H]+	2.25	1.9	0.18	1918407
META3656	MCy9003	[M+H]+	1.99	1.68	0.01	1508559
META3666	MCy9003	[M+H]+	1.99	1.68	0.01	1508559
META2894	MCy10674	[M+H]+	2.08	1.75	0.02	1500215
META3380	MCy11382	[M+H]+	1.39	1.17	0.03	1494407
META3384	MCy11382	[M+H]+	2.34	1.98	0.06	1439725
META3379	MCy11382	[M+H]+	1.91	1.61	0.06	1415324
META3798	MCy11957	[M+H]+	1.24	1.04	0.03	1412989
META3627	MCy11770	[M+H]+	1.69	1.42	0.01	1190123
META3792	MCy11957	[M+H]+	2.19	1.85	0.02	1184812
META3795	MCy11957	[M+H]+	2.75	2.32	0.03	1177042

META3630	MCy11770	[M+H]+	1.88	1.59	0.02	1174415
META3626	MCy11770	[M+H]+	1.51	1.27	0.01	1141921
META3654	MCy11812	[M+H]+	2.16	1.82	0	1105433
META3664	MCy11812	[M+H]+	2.16	1.82	0	1105433
META3797	MCy11957	[M+H]+	1.53	1.29	0.03	1101673
META3382	MCy11382	[M+H]+	2.26	1.91	0.04	1101512
META3793	MCy11957	[M+H]+	2	1.69	0.04	1091190
META3625	MCy11770	[M+H]+	1.34	1.13	0.02	1063321
META3800	MCy11957	[M+H]+	2.3	1.94	0.03	1050810
META3652	MCy11812	[M+H]+	1.97	1.67	0	1000296
META3662	MCy11812	[M+H]+	1.97	1.67	0	1000296
META3386	MCy11382	[M+H]+	1.83	1.54	0.05	960435
META3632	MCy11561	[M+H]+	1.89	1.6	0.17	916481
META2866	MCy9539	[M+H]+	2.94	2.48	0	901179
META3535	MCy10644	[M+H]+	1.55	1.31	0	732447
META3381	MCy11382	[M+H]+	2.02	1.7	0.05	702291
META3801	MCy11957	[M+H]+	1.76	1.48	0.03	655294
META2864	MCy9537	[M+H]+	2.88	2.43	0.21	612085
META2862	MCy9534	[M+H]+	3.3	2.78	0.18	605052
META2898	MCy10743	[M+H]+	2.55	2.15	0.14	590448
META3628	MCy11770	[M+H]+	2.3	1.94	0.01	544501
META3377	MCy11661	[M+H]+	1.77	1.5	0.1	529677
META906	MCy10978	[M+H]+	3.29	2.78	0.01	507808
META3531	MCy10644	[M+H]+	1.89	1.59	0	493598
META2865	MCy9538	[M+H]+	2.82	2.38	0.01	481301
META3368	MCy11661	[M+H]+	2.05	1.73	0.1	480084
META3387	MCy11382	[M+H]+	2.34	1.98	0.05	470028
META3378	MCy11382	[M+H]+	2.62	2.21	0.05	451114
META3719	MCy11910	[M+H]+	2.49	2.1	0.01	437266
META3773	MCy11951	[M+H]+	2.68	2.26	0.18	410320
META908	MCy10981	[M+H]+	2.63	2.22	0.02	386870
META3385	MCy11382	[M+H]+	2.55	2.16	0.04	358105
META3007	MCy9003	[M+H]+	4.43	3.74	0.03	349720
META3653	MCy11812	[M+H]+	2.19	1.85	0	342958
META3663	MCy11812	[M+H]+	2.19	1.85	0	342958
META2914	MCy9074	[M+H]+	3.16	2.67	0.02	333870
META3537	MCy10644	[M+H]+	1.77	1.49	0	332478
META3796	MCy11957	[M+H]+	3.19	2.69	0.03	331030
META3375	MCy11661	[M+H]+	2.26	1.91	0.09	330665
META3665	MCy11812	[M+H]+	2.03	1.71	0	297483
META3655	MCy11812	[M+H]+	2.03	1.71	0	297483
META907	MCy10979	[M+H]+	3.53	2.98	0.17	294340
META916	MCy10699	[M+H]+	3.32	2.8	0.01	283347
META2900	MCy10935	[M+H]+	3.04	2.57	0.01	266416

META3774	MCy11952	[M+H]+	1.67	1.41	0.18	249266
META2855	MCy10703	[M+H]+	3.29	2.78	0.03	245618
META3010	MCy9003	[M+H]+	4.05	3.41	0.02	242014
META2861	MCy9482	[M+H]+	3.39	2.86	0.2	240527
META3501	MCy11668	[M+H]+	1.4	1.18	0.14	237373
META3534	MCy10644	[M+H]+	1.99	1.68	0	234693
META904	MCy10954	[M+H]+	3.11	2.62	0.02	233646
META3598	MCy11666	[M+H]+	1.99	1.68	0.14	224143
META3370	MCy11661	[M+H]+	2.11	1.78	0.1	220431
META2917	MCy9156	[M+H]+	3.22	2.71	0.11	219457
META3017	MCy9003	[M+H]+	3.35	2.83	0.02	216502
META192	Mxf412	[M+H]+	3.33	2.81	0.26	213502
META1688	Mxf253	[M+H]+	4.09	3.45	0.24	212913
META2228	Mxf144	[M+H]+	3.28	2.77	0.07	205143
META2826	MCy9070	[M+H]+	4.48	3.78	0.16	202006
META2863	MCy9536	[M+H]+	3.01	2.54	0.01	201844
META3816	MCy11987	[M+H]+	1.53	1.29	0.03	196113
META3516	MCy11308	[M+H]+	1.72	1.45	0	195987
META3549	MCy10732	[M+H]+	1.7	1.43	0.15	195494
META3008	MCy9003	[M+H]+	3.35	2.82	0.03	190160
META3636	MCy11574	[M+H]+	3	2.53	0.15	187742
META910	MCy10982	[M+H]+	4.37	3.68	0.02	184026
META332	Mxf401	[M+H]+	2.87	2.42	0.25	183405
META3533	MCy10644	[M+H]+	1.79	1.51	0.01	183224
META925	MCy9541	[M+H]+	3.74	3.16	0.02	181563
META3383	MCy11382	[M+H]+	1.54	1.3	0.05	176591
META3009	MCy9003	[M+H]+	3.66	3.08	0.03	172619
META2854	MCy10700	[M+H]+	4.09	3.45	0.03	163946
META3548	MCy10732	[M+H]+	1.87	1.58	0.15	160908
META3545	MCy10732	[M+H]+	2.07	1.75	0.14	158985
META2895	MCy10717	[M+H]+	2.4	2.02	0.15	154452
META2824	MCy9067	[M+H]+	3.93	3.31	0.01	149721
META3411	MCy11647	[M+H]+	2.47	2.08	0.09	148749
META924	MCy9540	[M+H]+	3.9	3.29	0.03	147972
META3515	MCy11305	[M+H]+	1.61	1.36	0.01	145717
META3634	MCy11564	[M+H]+	2.02	1.71	0.15	144130
META3605	MCy11666	[M+H]+	2.5	2.11	0.15	142779
META2901	MCy10948	[M+H]+	3.85	3.25	0.02	142519
META3659	MCy11812	[M+H]+	3.04	2.57	0	141326
META3649	MCy11812	[M+H]+	3.04	2.57	0	141326
META3564	MCy11306	[M+H]+	2.04	1.72	0	138410
META3635	MCy11571	[M+H]+	2.97	2.51	0.16	138077

3 *Corallococcus sp.* MCy9049 and *Myxococcaceae sp.* MCy9003 Sequence Analysis

3.1 Preparation of genomic DNA for Genome sequencing

To isolate total DNA for sequencing purposes such as PacBio sequencing, phenol-chloroform gDNA extraction is used.

1) Spin down 50 mL of fresh myxobacterial culture 6000 rcf 10 min
2) Discard the supernatant
3) Wash the cells once with SET Buffer, centrifuge at 6000 rcf 10 min
4) Resuspend cell pellet in 5 mL SET Buffer
5) Add 100 µL of lysozyme (50 mg/mL in ddH_2O) stock solution as well as 50 µL RNAse A (10 mg/mL in ddH_2O) stock solution
6) Add 300 µL Proteinase K solution (10 mg/mL 50 mM Tris 1 mM $CaCl_2$) invert several times and add 600µL 10% SDS solution
7) Incubate at 55 °C for 2 h, invert every 15 min
8) Add even Volume (6 mL) of Phenol/Chloroform/Isoamylalcohol (25:24:1) and swing the tube for 60 min at 5 rpm
9) Centrifuge the mixture at 6000 rcf for 5 min at room temperature
10) Transfer the upper phase into a new tube using a cut end 1 mL tip
11) Add even Volume (6 mL) of Phenol/Chloroform/Isoamylalcohol (25:24:1) and swing the tube for 60 min at 5 rpm
12) Centrifuge the mixture at 6000 rcf for 5 min at room temperature
13) Transfer the upper phase into a new tube using a cut end 1 mL tip
14) Add even Volume (6 mL) of Chloroform/Isoamylalcohol (24:1) and swing the tube for 60 min at 5 rpm
15) Centrifuge the mixture at 6000 rcf for 5 min at room temperature
16) Transfer the upper phase into a new tube using a cut end 1 mL tip

17) Add 1/10 of the total volume of 3 M NaOAc solution pH 5.5 and mix well by inverting several times
18) Add 2.5 Volumes of ice-cold ethanol (100% technical purity, -20 °C) and invert the tube several times, DNA precipitation should be visible as a cotton like fog in the tube
19) Spool the DNA on a sealed Pasteur pipette
20) Rinse the DNA with 70% Ethanol (cold, -20 °C)
21) Air dry the DNA for at least 15 minutes (Dry DNA will become completely translucid)
22) Resuspend dried DNA in 0.5 mL of ddH_2O and keep the Eppendorf tube at room temperature for 24 Hours

3.2 Results of Genome sequencing of MCy9049 and MCy9003 wild type

3.2.1 Sequencing results of *Corallococcus sp.* MCy9049

The genome of *Corallococcus sp.* MCy9049 was sequenced using illumina sequencing at GMAK at the Helmholtz institute for infectious diseases in Brunswick. Assembly of the sequence reads led to the creation of a fragmented genome sequence consisting of 17 contigs with a total length of 9,876,717 bp. It should be noted that this is only a rough estimate of the genome size of MCy9049 as there may be parts of the genome that have not been covered in these runs.

3.2.2 Sequencing results of *Myxococcaceae sp.* MCy9003

The wild type strain MCy9003 was sequenced using a PacBio RS II device at the DSMZ using a single SMRT cell. The raw sequence reads were assembled in the SMRT portal software as recommended by the manufacturer. The MCy9003 genome sequence consists of a single unitig representing the closed circular bacterial chromosome spanning 11,262,112 bp.

3.3 *In-silico* blast analysis of the myxoquinone biosynthetic gene cluster

Every coding sequence in the myxoquinone biosynthesis gene cluster was extracted translated and searched with the blastp algorithm against the RefSeq non-redundant protein sequence database available online at NCBI [5].

Table S5. *Tabulated blastP results for the CDS regions present in the myxoquinone biosynthetic gene cluster from* Corallococcus sp. *MCy9049*

CDS Name	Length [AA]	Closest homologue [Organism of origin]	Identity [%] and alignment length [AA]	Proposed function	Accession Nr.
mxqA	268	phospho-2-dehydro-3-deoxyheptonate aldolase [Myxococcus xanthus DK 1622]	80.2 / 267	DAHP synthase	ABF86718
mxqB	229	ubiquinone/menaquinone biosynthesis methyltransferase [Corallococcus macrosporus]	89.5 / 222	MenG type methyl transferase	AEI67012
mxqC	199	putative shikimate kinase [Myxococcus xanthus DK 1622]	74.0 / 150	shikimate kinase from the shikimate pathway	ABF86999
mxqD	415	putative shikimate 5-dehydrogenase [Myxococcus xanthus DK 1622]	66.0 / 207	shikimate 5 dehydrogenase from the shikimate pathway	ABF86855
mxqE	339	chorismate synthase [Myxococcus fulvus 124B02]	94.4 / 338	chorismate synthase	AKF81877
mxqF	356	3-dehydroquinate synthase [Myxococcus xanthus DK 1622]	85.2 / 326	DHQ synthase	ABF93127
mxqG	441	3-phosphoshikimate 1-carboxyvinyltransferase [Corallococcus macrosporus]	86.8 / 433	EPSPS synthase	AEI67007
mxqH	434	Citrate synthase [Chloroflexi bacterium]	85.6 / 431	citrate synthase	HDY14418
mxqI	431	Isochorismate synthase [Myxococcus fulvus 124B02]	86.3 / 430	MenF like isochorismate synthase	AKF81872
mxqJ	589	2-succinyl-5-enolpyruvyl-6-hydroxy-3-cyclohexene-1-carboxylate synthase [Myxococcus fulvus 124B02]	87.8 / 588	MenD type SEPHCHC synthase	AKF81871
mxqK	283	2-succinyl-6-hydroxy-2,4-cyclohexadiene-1-carboxylate synthase [Corallococcus sp. ZKHCc1 1396]	86.7 / 280	MenH type SHCHC	WP_193430157
mxqL	306	1,4-Dihydroxy-2-naphtoate prenyltransferase [Roseiflexus sp. RS-1]	74.4 / 292	MenA type prenyl transferase	ABQ89887
mxqM	384	o-succinylbenzoate synthase [Chloroflexi bacterium]	74.0 / 353	MenC type aromatase	HEM20448
mxqN	487	O-succinylbenzoate-CoA ligase [Myxococcus fulvus 124B02]	72.2 / 358	MenE like CoA ligase	AKF81867
mxqO	457	Cytochrome P450 family protein [Corallococcus coralloides DSM 2259]	96.3 / 440	putative myxoquinone hydroxylase	AFE08485

3.4 Tabulated overview over the positions of the mxq BGC in strains with published genomes

The following table contains the regions that were extracted for alignment of the *mxq* clusters from the publicly available *Cystobacterinae* genomes that were analysed.

Table S6. Myxobacterial type strains, whose genomes contain the myxoquinone biosynthetic gene cluster (sequences accessible in the NCBI non redundant (nr) database).

Species Name	Strain	GenBank Accession no.	BGC Locus [bp]
Myxococcus xanthus	DK1622	NC_008095	4089344-4124016
Stigmatella aurantiaca	DW 4/3-1	NC_014623	4883536-4918458
Myxococcus macrosporus	HW-1	CP002830	6030378-6065326
Corallococcus coralloides	DSM 2259	NC_01730	5705590-5738257
Myxococcus fulvus	124B02	CP006003	6063603-6099479
Archangium gephyra	DSM 2261	CP011509	6235954-6268590
Cystobacter fuscus	DSM 52655	CP022098	5642534-5676764
Melittangium boletus	DSM 14713	NZ_CP022163	8328959-8363946
Corallococcus macrosporus	DSM 14679	NZ_CP022203	4128546-4162061
Corallococcus coralloides	B035	NZ_CP034669	5442043-5475834
Archangium violaceum	SDU34	NZ_CP069338	9280257-9314816

The different *mxq* clusters are analyzed with the clinker tool to perform a multigene analysis, which highlights the clusters to be rather conserved among *Cystobacterinae* [6].

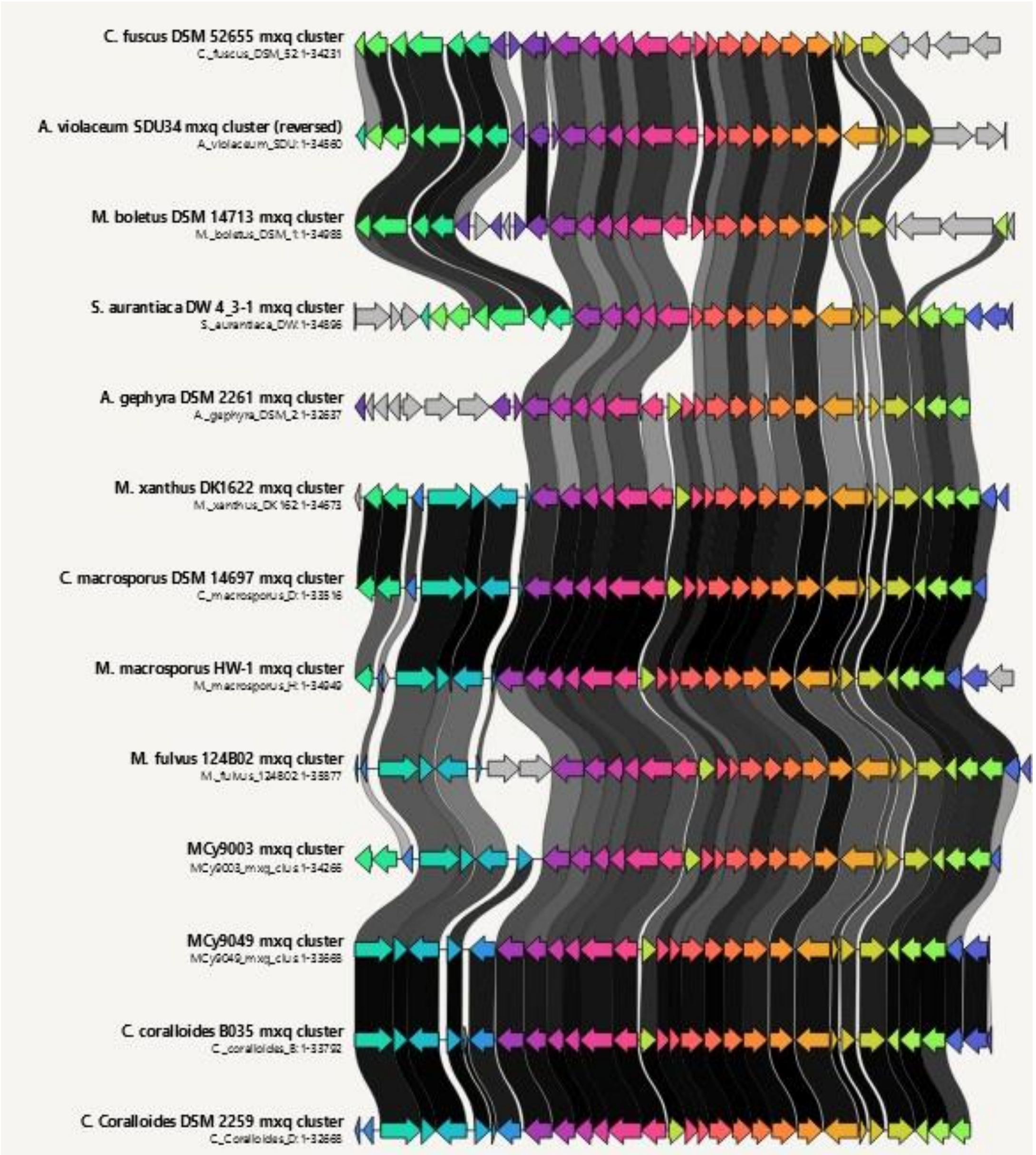

Figure S1. *Clinker based comparison of the* mxq *Biosynthetic gene cluster regions that reveals the core BGC to be highly conserved among* Cystobacterinae*.*

4 Isolation and structure elucidation of four new Myxoquinones

4.1 Fermentation, isolation and purification of the Myxoquinones

4.1.1 Cultivation and extraction of Myxoquinone 739 and 825 from MCy9049

The *Corallococcus* sp. strain MCy9049 is fermented in 50 mL PYGS medium as a seed culture flask on an Orbiton shaker at 160 rpm and 30 °C. The white culture medium becomes translucid and ball shaped myxobacterial biofilm clumps appear after 7 to 11 days of fermentation. This pre-culture is used to inoculate 6 x 2 L PYGS medium supplemented with 2 % XAD-16 resin suspension in sterilized water in 6 x 5 L baffled shake flasks on an Orbiton shaker at 160 rpm and 30 °C. Fermentation is complete after 16 days. Cells and XAD-16 resin are harvested by centrifugation on a Beckmann Avanti J-26 XP with the JLA 8.1 rotor at 6000 rcf. Combined resin and cells are freeze dried and subsequently extracted using 2x 500 mL of a 2:1 mixture of methanol and chloroform. The combined extracts are concentrated on a rotary evaporator and partitioned between methanol and hexane. The myxoquinones remain in the methanol phase. The methanol phase is dried with a rotary evaporator and the residue is partitioned between water and chloroform. The myxoquinones are retained in the chloroform phase. The chloroform phase is concentrated and stored in an air-tight glass vial at -20 °C until further processing.

4.1.2 Purification of Myxoquinone 739 and 825

Purification of myxoquinone 739 and 825 is carried out using a Dionex Ultimate 3000 SDLC low pressure gradient system on a Waters Acquity CSH C18 250x10mm 5µm column with the eluents H_2O +0.1% FA as A and ACN + 0.1% FA as B, a flow rate of 5 mL/min and a column thermostatic at 30 °C. myxoquinone A and B are detected by UV absorption at 270 nm and purification is done by time dependent fraction collection. Separation is started with a plateau at 95% A for 2 minutes followed by a ramp to 5% A during 24 minutes. The A content is kept at 5% A for 2 minutes. The A content is ramped back to starting conditions during 30 seconds and the column is re equilibrated for 2 minutes. After evaporation, the myxoquinones 739 and 825 are obtained as pale yellowish amorphous solids. The compounds are dried and stored in an air-tight glass vial at -20 °C.

4.1.3 Cultivation and extraction of Myxoquinone 843 and 861 from MCy9003

The *Myxococcaceae sp.* strain MCy9003 is fermented in 50 mL CyHv3 medium as a seed culture flask on an Orbiton shaker at 160 rpm and 30 °C. The white culture medium becomes translucid and ball shaped myxobacterial biofilm clumps appear after 7 to 11 days of fermentation. This pre-culture is used to inoculate 6 x 2 L CyHv3 medium supplemented with 2 % XAD-16 resin suspension in sterilized water in 6 x 5 L baffled shake flasks on an Orbiton shaker at 160 rpm and 30 °C. Fermentation is complete after 16 days. Cells and XAD-16 resin are harvested by centrifugation on a Beckmann Avanti J-26 XP with the JLA 8.1 rotor at 6000 rcf. Adsorber resin XAD-16 and cells from a 5 x 2 L culture of MCy9003 were freeze dried and extracted three times with 150 ml acetone. The dried extract was redissolved in 1 ml MeOH and centrifuged with 21.500 g for 10 min. The supernatant was fractionated using size exclusion chromatography with Sephadex LH 20 resin with MeOH as eluent with a flow rate of 12 drops / min. 420 drops were collected for every fraction. An aliquot of every fraction screened for the target molecules using LC-MS. The fractions containing the target masses were combined and dried. The residue was dissolved in MeOH. The target molecules myxoquinone 843 and myxoquinone 861 were isolated by semi preparative reverse phase chromatography. Following setup was used: Dionex Ultimante 3000; H_2O + 0.1 % FA as eluent A and ACN + 0.1 % FA as eluent B; Phenomenex Jupiter Proteo 250 x 10 mm, 4 µm, 30 Å column; flow rate 6 ml/min; Following gradient was applied: 0-2.5 min: 5 % eluent B; 2.5 -22.5 min linear increase of eluent B to 95 %; 22.5 – 25 min 95 % eluent B; 25-26 min linear decrease of eluent B to 5 %; 26-31 min re-equilibration with 5 % eluent B. Myxoquinone 861 was collected from 16.25-16.55 min and myxoquinone 843 was collected from 19.1-19.3 min. After evaporation, the myxoquinones 843 and 861 are obtained as pale yellowish amorphous solids. The compounds are dried and stored in an air-tight glass vial at -20 °C.

4.2 UV spectra of the Myxoquinones

The myxoquinones 739 and 825 display an intriguing UV spectrum with four UV maxima at 220 nm, 242 nm, 270 nm and 332 nm. The complete UV spectrum acquired in acetonitrile of myxoquinone 739 depicted below.

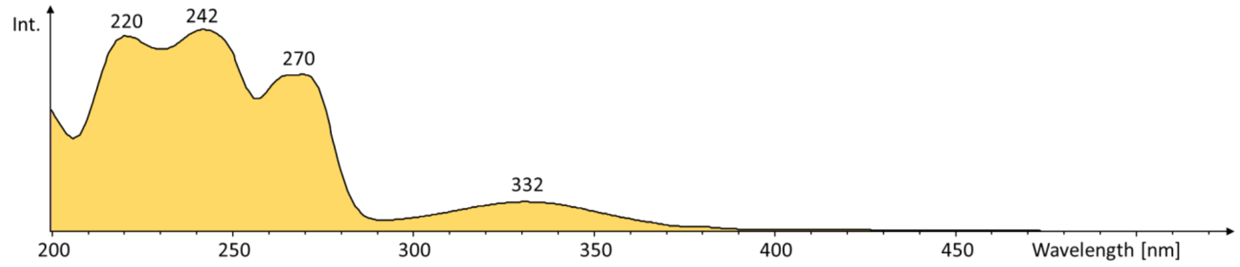

Figure S2. *UV spectrum of myxoquinone 739 in Acetonitrile*

The myxoquinones 861 and 843 display an intriguing UV spectrum with three UV maxima at 222 nm, 255 nm and 305 nm. It is worth noting that the overall UV activity of myxoquinone 843 and 861 is reduced as the chromophore is smaller due to hydration of the naphthoquinone ring. The complete UV spectrum acquired in acetonitrile of myxoquinone 843 depicted below.

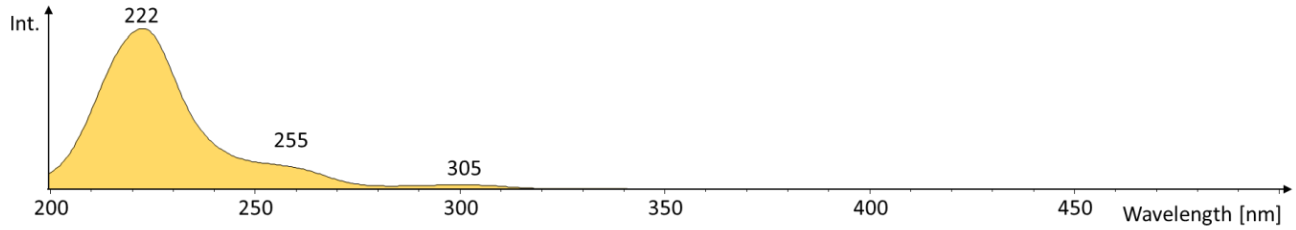

Figure S3. *UV spectrum of myxoquinone 843 in Acetonitrile*

4.3 Tandem MS spectra of the myxoquinones

Tandem MS spectra were acquired based on our UHPLC-hrMS qTOF system according to the methods described in the analytical methods section. A representative set of tandem MS spectra is presented here that visualizes the structural relatedness of the myxoquinones on a MS² spectral basis due to the general similarity in the fragmentation pattern as well as the common MS fragment 187.075 Da.

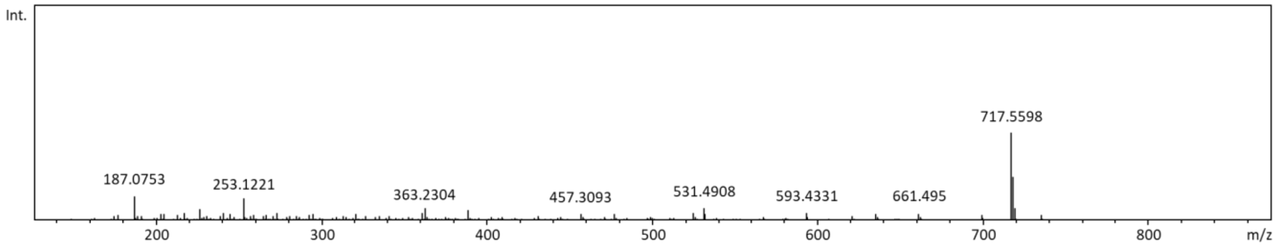

Figure S4. *Tandem MS spectrum of myxoquinone 825 acquired on a Bruker maXis 4G qTOF spectrometer*

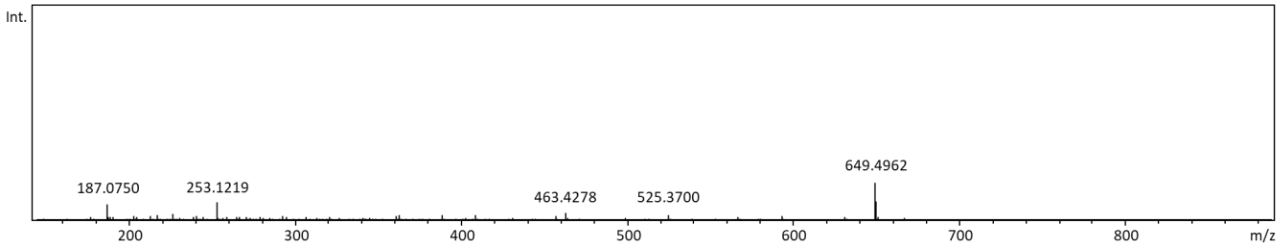

Figure S5. *Tandem MS spectrum of myxoquinone 739 acquired on a Bruker maXis 4G qTOF spectrometer*

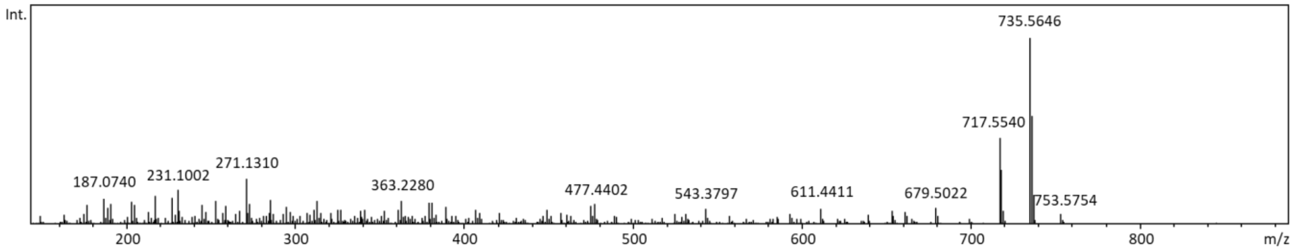

Figure S6. *Tandem MS spectrum of myxoquinone 861 acquired on a Bruker maXis 4G qTOF spectrometer*

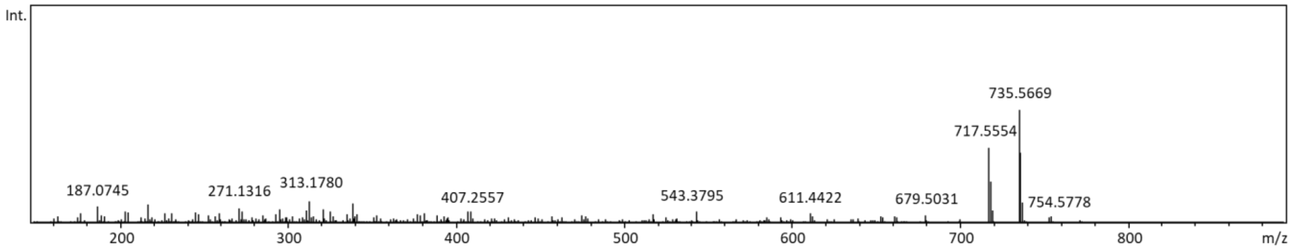

Figure S7. *Tandem MS spectrum of myxoquinone 843 acquired on a Bruker maXis 4G qTOF spectrometer*

4.4 NMR based structure elucidation

4.4.1 NMR conditions and spectroscopic data

1D and 2D NMR data used for structure elucidation of the myxoquinone derivatives is acquired in $CDCl_3$ on a Bruker Ascend 700 spectrometer equipped with a TCI 5mm probe head (^{1}H at 700 MHz, ^{13}C at 175 MHz) and Bruker Ultra Shield 500 spectrometer equipped with a TCI 5mm probe head (^{1}H at 500 MHz, ^{13}C at 125 MHz). All observed chemical shift values (δ) are given in in ppm and coupling constant values (J) in Hz. Standard pulse programs were used for HMBC, HSQC, gCOSY and DQF COSY experiments. HMBC experiments were optimized for $^{2,3}J_{C\text{-}H}$ = 6 Hz. The spectra were recorded in $CDCl_3$ and chemical shifts of the solvent signals at δ^H 7.27 ppm and δ^C 77.0 ppm were used as reference signals for spectra calibration. To increase sensitivity, the measurements were conducted in a 5 mm Shigemi tube (Shigemi Inc., Allison Park, PA 15101, USA). The NMR signals are grouped in the tables below and correspond to the numbering

in the schemes corresponding to every table. All structure formulae devised by NMR will be made publicly available under their corresponding name in NPatlas. [7,8]

4.4.2 Structure elucidation of the Myxoquinone derivatives

4.4.2.1 Myxoquinone 843

Myxoquinone 843 **(1)** was isolated as a yellow solid. The high resolution mass spectrum showed an intense signal for the single charged ion $[M+H]^+$ at 843.634 *m/z*. Using Bruker data analysis 4.4 software, the sum formula of $C_{51}H_{86}O_9$ was determined (calc. 843.635 measured 843.634 Δ = 1.2 ppm).

The proton spectra of **1** exhibited four signals at δ_{H8} 8.12, δ_{H5} 8.06, δ_{H6} 7.79 and δ_{H7} 7.79 characteristic for methine protons inside an aromatic ring system. In addition, two signals with characteristic shift values for olefinic protons can be found at $\delta_{H2'}$ 5.35 and δ_{H8} 5.09. Furthermore, a methine proton at δ_{H8} 3.06, two proton signals from a diastereotopic methylene protons at $\delta_{H1'a}$ 2.60 and $\delta_{H1'b}$ 2.71 and three signals with characteristic resonances of methylene protons at $\delta_{H5'}$ 2.09, $\delta_{H4'}$ 2.02 and $\delta_{H8'}$ 1.95 can be found. Moreover, two distinct proton signals are located up field shifted at $\delta_{H33'}$ 1.71 and $\delta_{H32'/40'}$ 1.23 in the proton spectrum, suggesting the presence of multiple methyl groups. Close inspection of the HSQC spectrum revealed one additional methylene group ($\delta_{C30'}$ 44.3, $\delta_{H30'}$ 1.45) and two broad intense methylene signals (δ_C 42.0-43.0, δ_H 1.42-1.47) and (δ_C 18.0-19.0, δ_H 1.42-1.47) indicating multiple methylene groups with comparable or identical chemical properties. Moreover, one additional methyl group ($\delta_{C2\ Me'}$ 22.3, $\delta_{H2\text{-}Me'}$ 1.31) and a broad intense methyl group signal (δ_C 26.5-27.4, δ 1.15-1.24) can be found.

Analysis of the COSY spectrum could show that the aromatic methine protons from H-5- H-8 form a spin system, similar to an ortho bis substituted benzene ring. In addition, the COSY data revealed the methine proton at δ_{H8} 3.06 is a vicinal coupling partner of the two diastereotopic methylene protons at $\delta_{H1'a}$ 2.60 and $\delta_{H1'b}$ 2.71, which shows further vicinal coupling to an olefinic proton at $\delta_{H2'}$ 5.35. Moreover, both methylene groups ($\delta_{C4'}$ 39.6, $\delta_{H4'}$ 2.02) and ($\delta_{C5'}$ 26.2, $\delta_{H5'}$ 2.09) form a linear spin system with a sp^2 hybridized methine group ($\delta_{C6'}$ 124.1, $\delta_{H6'}$ 5.09), as indicated by the corresponding COSY cross peaks.

Inspection of the HMBC spectra revealed further structural information: The aromatic proton at δ_{H8} 8.12 showed HMBC correlations to a carbon resonance at δ_{C8a} 136.0 and δ_{C1} 200.6. In addition, the other aromatic proton on the opposite side of the spin system at δ_{H5} 8.06 showed HMBC correlations to two carbon resonances at δ_{C4a} 131.6 and δ_{C4} 195.9, suggesting a naphthoquinone like scaffold. Close inspection of the HMBC spectrum revealed HMBC correlations from the methine proton at δ_{H8} 3.06 to the same carbon resonance at δ_{C4} 195.9, a carbon resonance to a quaternary carbon at δ_{C2} 78.2 and to a methyl group ($\delta_{C2\text{-}Me}$ 22.3, $\delta_{H2\text{-}Me}$ 1.31). In return, this methyl group shows HMBC correlations to the same

quaternary carbon at δ_{C2} 78.2 and to δ_{C1} 200.6. These characteristic shift values and correlations suggest a 2-methyl, 2-hydroxy naphtoquinone scaffold. As indicated by the previously described COSY correlations, position 3 is linked to a diastereotopic methylene group and a sp^2 hybridized methine group. Both diastereotopic methylene protons at $\delta_{H1'a}$ 2.60 and $\delta_{H1'b}$ 2.71 show HMBC correlations to carbon resonance of a quaternary carbon at $\delta_{C3'}$ 136.6. Outgoing from the proton resonance of a methyl group at $\delta_{H33'}$ 1.71, a correlation to the same resonance, to the carbon resonance of a methine group ($\delta_{C2'}$ 122.3, $\delta_{H2'}$ 5.35) and to the resonance of another methine group at ($\delta_{C4'}$ 39.6, $\delta_{H4'}$ 2.02) could be observed. This specific pattern of COSY and HMBC correlations implies the methyl group is attached to a quaternary carbon, linked to methine group via a double bond, like in a prenyl unit. In addition, another methylene group is linked to the sp^2 hybridized quaternary carbon. Previously described COSY correlations and HMBC correlations from the methylene proton at $\delta_{H5'}$ 2.09 and protons of another methyl group at $\delta_{H34'}$ 1.58 to the carbon resonance at $\delta_{C6'}$ 136.6 strongly imply a similar prenyl type scaffold.

A group of overlapping methyl groups at (δ_C 26.5-27.4, δ 1.15-1.24) show HMBC correlations to a carbon resonance between δ_C 72.0-73.0 and to a group of overlapping methylene signals at (δ_C 42.0-43.0, δ_H 1.42-1.47) and (δ_C 18.0-19.0, δ_H 1.42-1.47). Considering the previously described prenyl scaffold, this suggests here the presence of a repetitive prenyl element that is hydroxylated at the quaternary carbon. In addition, this structural proposal is supported by HMBC correlations from a two identical methyl group at ($\delta_{C32',40'}$ 29.1, $\delta_{H32',40'}$ 1.23) to a distinct carbon resonance of a quaternary carbon at $\delta_{C31'}$ 70.7 and a methylene group at ($\delta_{C30'}$ 44.3, $\delta_{H30'}$ 1.47). The small deviation of the shift values, compared to the repetitive hydroxylated prenyl unit, imply this scaffold to be the end of a chain of hydroxylated prenyl unit. Considering the molecular sum formula and the described structural scaffolds the structure is comparable to a quinone with 8 prenyl units. The differences are the hydroxylation of the naphthoquinone ring and at the last six prenyl units.

4.4.2.2 Myxoquinone 861

Myxoquinone 861 **(2)** was isolated as a yellow solid. The high resolution mass spectrum showed an intense signal for the single charged ion $[M+H]^+$ at 861.653 *m/z*. Using Bruker data analysis 4.4 software, the sum formula of $C_{51}H_{88}O_{10}$ was determined (calc. 861.650 measured 861.653 Δ = 3.5 ppm). The proton spectrum of myxoquinone 861 strongly resembled the spectrum of **1**. The most obvious different to be found was the presence of only one olefinic proton at $\delta_{H2'}$ 5.35 in contrast to **1**. The *hr*MS derived sum formula suggests an additional hydroxyl group. Considering the presence of only one olefinic proton, this finding implies seven prenyls unit to be hydroxylated, in contrast to **1**.

4.4.2.3 Myxoquinone 825

Myxoquinone 825 **(3)** was isolated as a yellow solid. The high resolution mass spectrum showed an intense signal for the single charged ion $[M+H]^+$ at 825.623 *m/z*. Using Bruker data analysis 4.4 software, the sum formula of $C_{51}H_{84}O_8$ was determined (calc. 825.624 measured 825.623 Δ = 1.21 ppm). The proton spectrum of myxoquinone 825 showed significant similarities to the spectrum of **1**. Differences to be found were in the area of aromatic proton signals: Here, only two multiplets at $\delta_{H7,6}$ 7.70 and $\delta_{H5,8}$ 7.70 were found. In addition, the signal of the methine proton of ring position 3 was missing and the signals of the diastereotopic methylene protons were shifted into a single methylene proton signal at $\delta_{H1'}$ 3.38 and the signal of the methyl protons from ring position 2 were downfield shifted to $\delta_{H2\ Me}$ 2.20. Inspection of the HMBC spectrum showed correlations from this methyl group to two quaternary carbon resonances at δ_{C2} 146.0 and δ_{C3} 143.2. Moreover, the same HMBC correlations can be observed from the first methylene protons of the first prenyl unit at $\delta_{H1'}$ 3.38. These findings and the *hr*MS obtained sum formula suggest an unhydroxalated methyl-naphtoquinone ring, like found in typical quinone scaffolds.

4.4.2.4 Myxoquinone 739

Myxoquinone 739 **(4)** was isolated as a yellow solid. The high resolution mass spectrum showed an intense signal for the single charged ion $[M+H]^+$ at 739.549 *m/z*. Using Bruker data analysis 4.4 software, the sum formula of $C_{46}H_{74}O_7$ was determined (calc. 739.551 measured 739.549 Δ = 2.7 ppm). The proton spectrum of myxoquinone 739 was identical with the spectrum of myxoquinone 825. Based on the *hr*MS derived sum formula, we propose an identical structure with on hydroxylated prenyl moiety less, compared to myxoquinone 825.

4.4.3 Tabulated NMR signals for every myxoquinone derivative

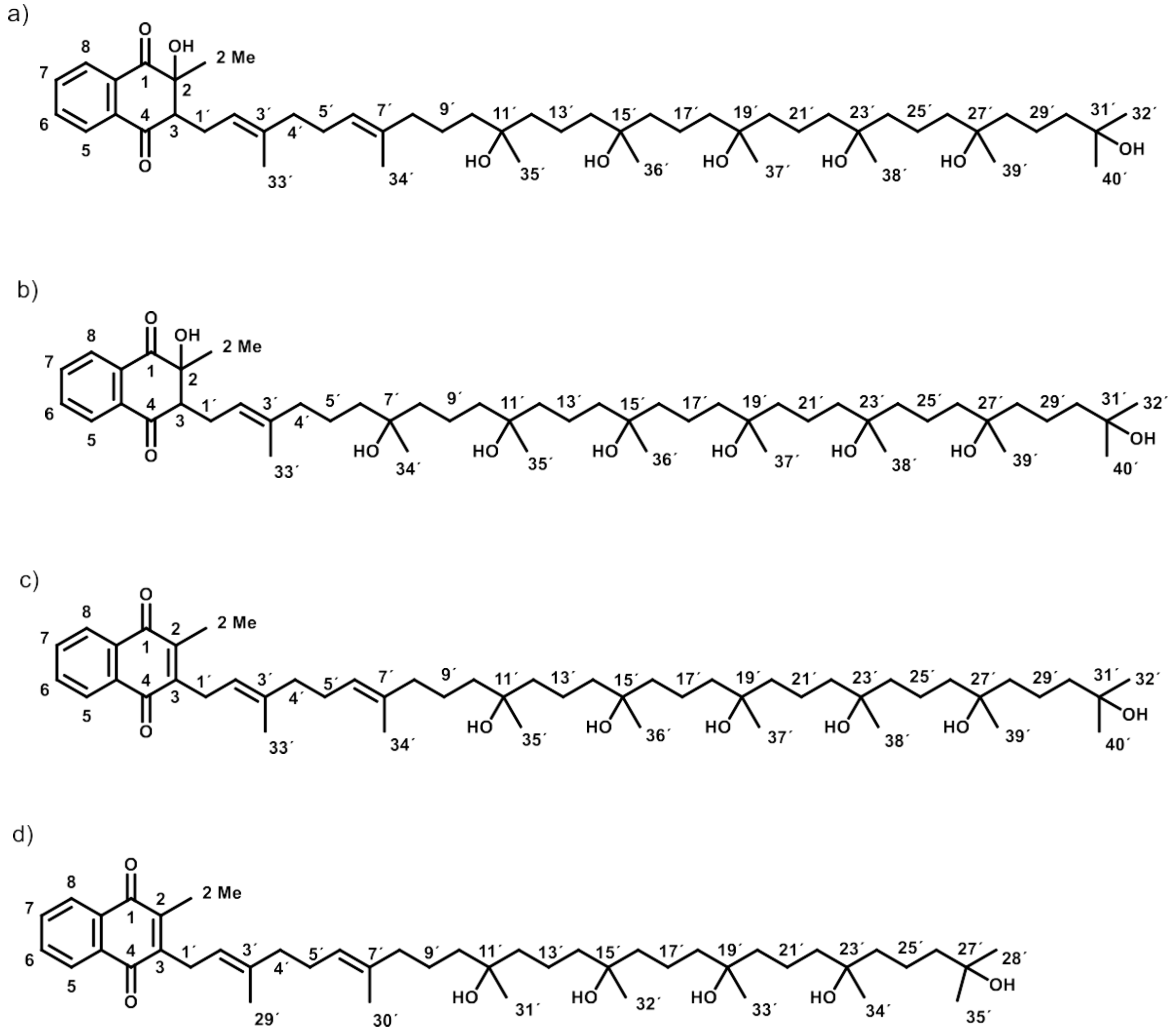

Figure S8. *Structures of isolated myxoquinone variants: a) myxoquinone 843, b) myxoquinone 861, c) myxoquinone 825 d) myxoquinone 739 including atom numbering correspondent to the NMR tables*

Table S7. *spectroscopic values of myxoquinone 843 acquired in $CDCl_3$ at 700 MHz*

position	$\delta^{13}C^a$	δ^1H^b	Multiplicity (J in Hz)	COSY[c]	HMBC[d]
1	200.6				
2	78.2				
2Me	22.3	1.31	m		3, 2
3	60.3	3.06	m		2Me, 2, 4
4	195.9				
4a	131.6				
5	126.3	8.06	m	7	4a, 6, 4
6	134.2	7.79	br d (1.71)		8, 8a
7	133.7	7.78	m	5, 8	4a
8	127.2	8.12	m	7	8a, 1
8a	136.0				
1´a	21.9	2.60	m	2´	3, 2, 2´, 3´, 4
1´b	21.9	2.71	m	2´	3, 2´, 3´, 4
2´	122.3	5.35	m	1´a, 1´b	33´, 1´, 4´, 3
3´	136.3				
4´	39.6	2.02	m	5´	33´, 5´, 2´, 6´, 3´
5´	26.2	2.09	br dd (14.64, 7.05)	6´,4´	4´, 6´
6´	124.1	5.09	m	5´	34´, 8´
7´	134.2				
8´	39.9	1.95	m	9´	
9´	18.4	1.41	m		
10´	42.0-43.0	1.42-1.47	m		
11´	72.0-73.0				
12´	42.0-43.0	1.42-1.47	m		
13´	18.0-19.0	1.42-1.47	m		
14´	42.0-43.0	1.42-1.47	m		
15´	72.0-73.0				
16´	42.0-43.0	1.42-1.47	m		
17´	18.0-19.0	1.42-1.47	m		
18´	42.0-43.0	1.42-1.47	m		
19´	72.0-73.0				
20´	42.0-43.0	1.42-1.47	m		
21´	18.0-19.0	1.42-1.47	m		

22´	42.0-43.0	1.42-1.47	m		
23´	72.0-73.0				
24´	42.0-43.0	1.42-1.47	m		
25´	18.0-19.0	1.42-1.47	m		
26´	42.0-43.0	1.42-1.47	m		
27´	72.0-73.0				
28´	42.0-43.0	1.42-1.47	m		
29´	18.0-19.0	1.42-1.47	m		
30´	44.3	1.47	m		29´
31´	70.7				
40´, 32´	29.1	1.23	m		30´, 31´
33´	16.1	1.71	s		4´, 2´, 3´
34´	15.3	1.58	br s		8´, 6´
35´ - 39´	26.5-27.1	1.15-1.24	m		

[a] acquired at 175 MHz and assigned from 2D NMR spectra, referenced to solvent signal $CDCl_3$ at δ 77.0 ppm.
[b] acquired at 700 MHz, referenced to solvent signal $CDCl_3$ at δ 7.27 ppm.
[c] proton showing COSY correlation to indicated proton.
[d] proton showing HMBC correlations to indicated carbons.

Table S8. *Spectroscopic values of myxoquinone 861 acquired in $CDCl_3$ at 700 MHz*

position	δ^{13}C[a]	δ^{1}H[b]	Multiplicity (J in Hz)	COSY[c]	HMBC[d]
1	200.5				
2	78.1				
2Me	22.7	1.31	s		3, 2, 1
3	60.2	3.06	dd (9.10, 2.69)	1´	2, 4
4	39.6	1.99	m	6´	1´, 5´, 6´, 2´, 3´
4a	131.3				
5	126.6	8.06	m	7, 6	4a, 7
6	134.7	7.79	m	5, 8	8, 8a
7	134.0	7.78	m	5, 8	4a
8	127.3	8.12	m	7, 6	
8a	136.2				
1´a	22.0	2.71	br d(15.00)	1´, 3, 2´	3, 2´, 3´, 4
1´b	22.0	2.60	m	1´, 2´	3, 2´, 3´, 4
2´	122.5	5.35	m	1´, 1´	
3´	136.5				

4´	39.6	1.99	m	6´	1´, 5´, 6´, 2´, 3´
5´	22.2	1.41	m		
6´	41.3	1.41	m	4´	
7´	72.5				
8´	42.0-43.0	1.42-1.47	m		
9´	18.0-19.0	1.42-1.47	m		
10´	42.0-43.0	1.42-1.47	m		
11´	72.0-73.0				
12´	42.0-43.0	1.42-1.47	m		
13´	18.0-19.0	1.42-1.47	m		
14´	42.0-43.0	1.42-1.47	m		
15´	72.0-73.0				
16´	42.0-43.0	1.42-1.47	m		
17´	18.0-19.0	1.42-1.47	m		
18´	42.0-43.0	1.42-1.47	m		
19´	72.0-73.0				
20´	42.0-43.0	1.42-1.47	m		
21´	18.0-19.0	1.42-1.47	m		
22´	42.0-43.0	1.42-1.47	m		
23´	72.0-73.0				
24´	42.0-43.0	1.42-1.47	m		
25´	18.0-19.0	1.42-1.47	m		
26´	42.0-43.0	1.42-1.47	m		
27´	72.0-73.0				
28´	42.0-43.0	1.42-1.47	m		
29´	18.0-19.0	1.42-1.47	m		
30´	44.0	1.47	m		
31´	70.7				
40´, 32´	29.1	1.23	s		30´, 31´
33´	16.1	1.70	m		4´, 2´
34´ - 39´	26.5-27.1	1.15-1.24	m		

[a] acquired at 175 MHz and assigned from 2D NMR spectra, referenced to solvent signal $CDCl_3$ at δ 77.0 ppm.
[b] acquired at 700 MHz, referenced to solvent signal $CDCl_3$ at δ 7.27 ppm.
[c] proton showing COSY correlation to indicated proton.
[d] proton showing HMBC correlations to indicated carbons.

Table S9. *Spectroscopic values of 825 acquired in $CDCl_3$ at 700 MHz*

position	$\delta^{13}C^a$	δ^1H^b	Multiplicity (J in Hz)	COSY[c]	HMBC[d]
1	185.3				
2	146.0				
2Me	12.5	2.20	m		3, 2, 1
3	143.2				
4	184.3				
4a	131.9				
5, 8	126.2	8.09	m	7, 6	4a, 8a, 7, 6, 4, 1
7, 6	133.3	7.70	m	5, 8	
8a	132.9				
1´	26.1	3.38	m	2´	2´, 3´, 3, 2, 4
2´	119.1	5.02	m	33´, 1´	4´, 2
3´	137.1				
4´	39.7	2.01	m	5´	5´, 2´, 6´, 3´
5´	26.3	2.08	br s	4´, 6´	6´
6´	124.1	5.06	td (6.92,6.92, 1.12)	34´, 5´	8´
7´	134.7				
8´	40.0	1.92	m		9´
9´	22.1	1.38	m		
10´	42.0-43.0	1.42-1.47	m		
11´	72.0-73.0				
12´	42.0-43.0	1.42-1.47	m		
13´	18.0-19.0	1.42-1.47	m		
14´	42.0-43.0	1.42-1.47	m		
15´	72.0-73.0				
16´	42.0-43.0	1.42-1.47	m		
17´	18.0-19.0	1.42-1.47	m		
18´	42.0-43.0	1.42-1.47	m		
19´	72.0-73.0				
20´	42.0-43.0	1.42-1.47	m		
21´	18.0-19.0	1.42-1.47	m		
22´	42.0-43.0	1.42-1.47	m		
23´	72.0-73.0				
24´	42.0-43.0	1.42-1.47	m		

25´	18.0-19.0	1.42-1.47	m		
26´	42.0-43.0	1.42-1.47	m		
27´	72.0-73.0				
28´	42.0-43.0	1.42-1.47	m		
29´	18.0-19.0	1.42-1.47	m		
30´	42.0-43.0	1.42-1.47	m		13´, 29´, 25´, 21´, 17´
31´	70.7				
40´, 32´	29.4	1.23	m		30´, 31´
33´	16.4	1.80	d (0.64)	2´	4´, 2´, 3´
34´	16.1	1.56	m	6´	8´, 6´, 7´
35´ - 39´	26.5-27.1	1.15-1.24	m		11´

[a] acquired at 175 MHz and assigned from 2D NMR spectra, referenced to solvent signal $CDCl_3$ at δ 77.0 ppm.
[b] acquired at 700 MHz, referenced to solvent signal $CDCl_3$ at δ 7.27 ppm.
[c] proton showing COSY correlation to indicated proton.
[d] proton showing HMBC correlations to indicated carbons.

Table S10. *Spectroscopic values of myxoquinone 739 acquired in $CDCl_3$ at 700 MHz*

Position	$\delta^{13}C^{a}$	$\delta^{1}H^{b}$	Multiplicity (J in Hz)	COSY[c]	HMBC[d]
1	185.0				
2	143.9				
2Me	13.0	2.20	m		2, 3, 4
3	146.6				
4	185.9				
4a	132.9				
5, 8	132.9	8.10	m	6, 7	8a, 4a, 6, 7, 1, 4
7, 6	133.7	7.70	m	5, 8	
8a	132.4				
1´	26.5	3.38	br d (6.95)	2´	2´, 3´, 2, 3, 1
2´	119.5	5.02	m	1´	29´, 5´, 4´, 3
3´	137.7				
4´	40.0	2.01	m		29´, 2´, 6´, 3´
5´	26.5	2.08	m	6´	4´, 6´, 7´, 3´
6´	124.5	5.06	m	5´	30´, 8´
7´	135.4				
8´	40.3	1.92	br s	9´	9´, 7´
9´	22.4	1.39	m	8´	

10´	42.0-43.0	1.42-1.47	m		
11´	72.5-73.5				
12´	42.0-43.0	1.42-1.47	m		
13´	18.0-19.0	1.42-1.47	m		
14´	42.0-43.0	1.42-1.47	m		
15´	72.5-73.5				
16´	42.0-43.0	1.42-1.47	m		
17´	18.0-19.0	1.42-1.47	m		
18´	42.0-43.0	1.42-1.47	m		
19´	72.5-73.5				
20´	42.0-43.0	1.42-1.47	m		
21´	18.0-19.0	1.42-1.47	m		
22´	42.0-43.0	1.42-1.47	m		
23´	72.5-73.5				
24´	42.0-43.0	1.42-1.47	m		
25´	18.0-19.0	1.42-1.47	m		
26´	44.7	1.47	m		
27´	71.3				
35´, 28´	29.6	1.23	s		25´,26´, 27´
31´-34´	26.5-27.4	1.15-1.24	m		

[a] acquired at 175 MHz and assigned from 2D NMR spectra, referenced to solvent signal $CDCl_3$ at δ 77.0 ppm.
[b] acquired at 700 MHz, referenced to solvent signal $CDCl_3$ at δ 7.27 ppm.
[c] proton showing COSY correlation to indicated proton.
[d] proton showing HMBC correlations to indicated carbons.

5 NMR spectra employed in myxoquinone structure elucidation

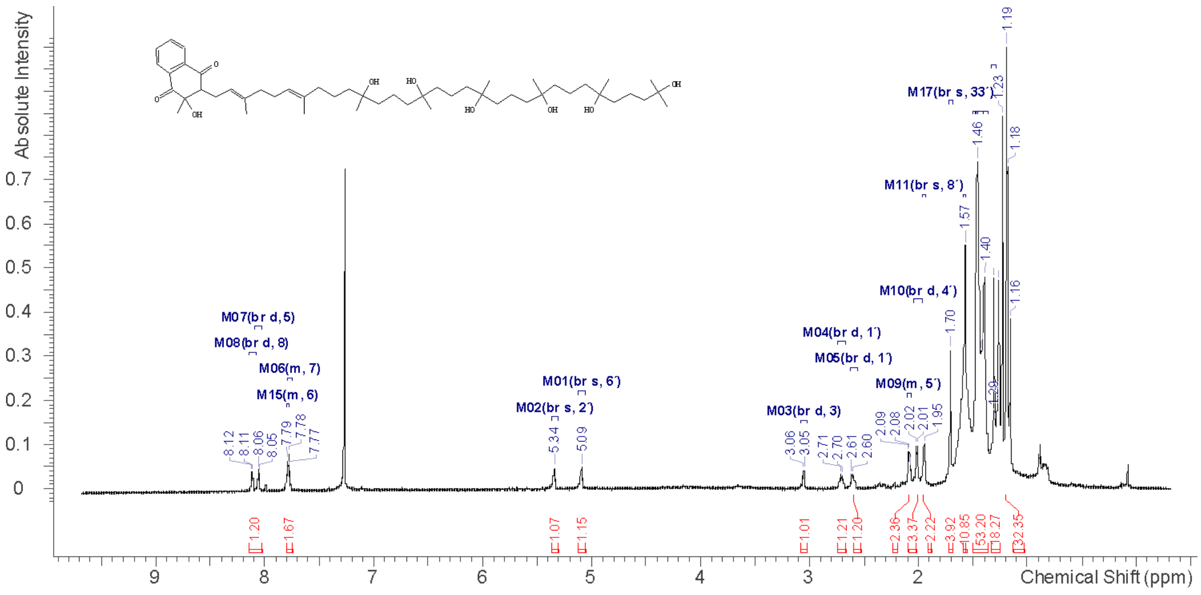

Figure S9. *^{1}H spectrum of myxoquinone 843 acquired in $CDCl_3$ at 700 MHz*

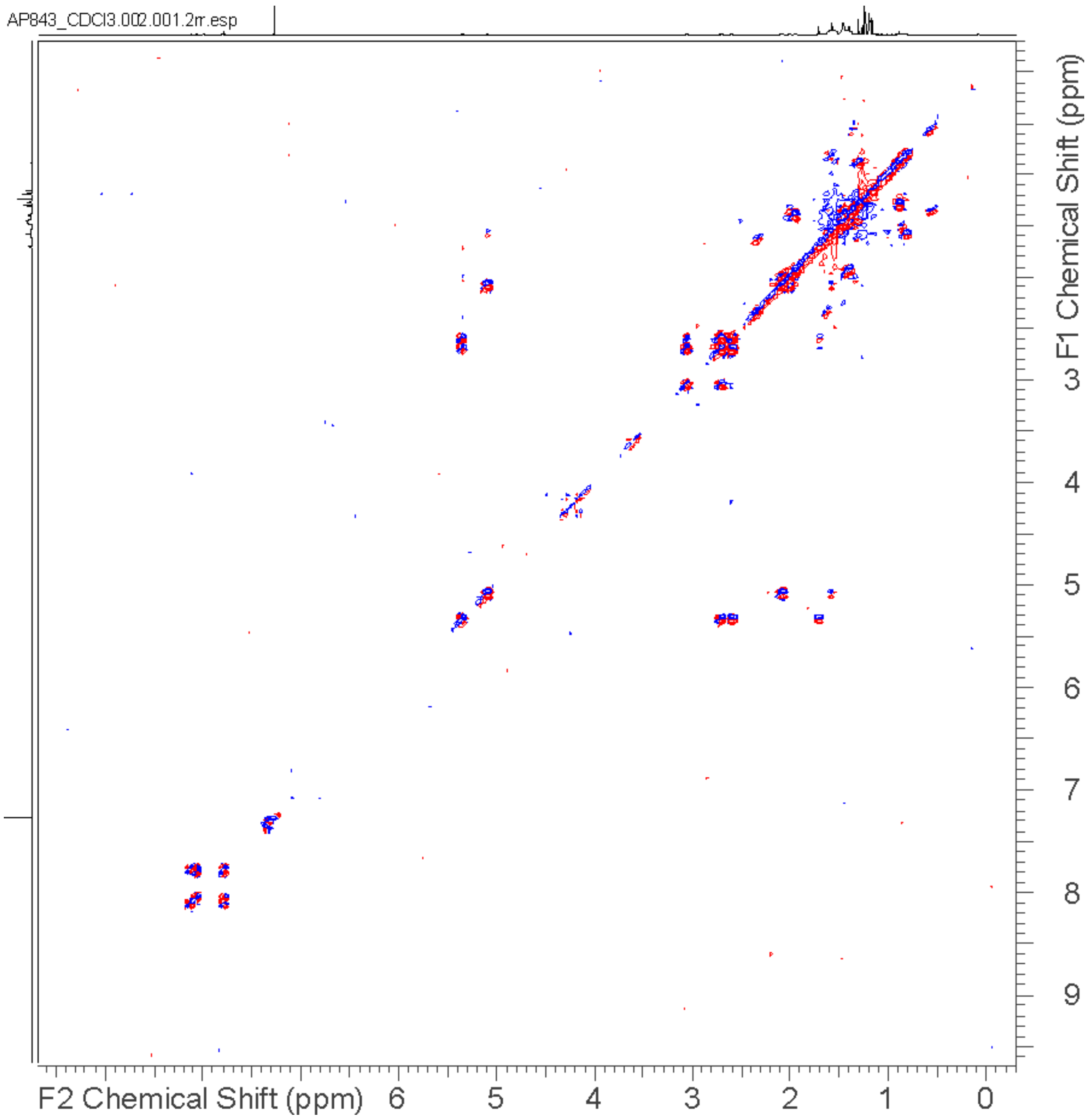

Figure S10. *DQF COSY spectrum of myxoquinone 843 acquired in $CDCl_3$ at 700 MHz*

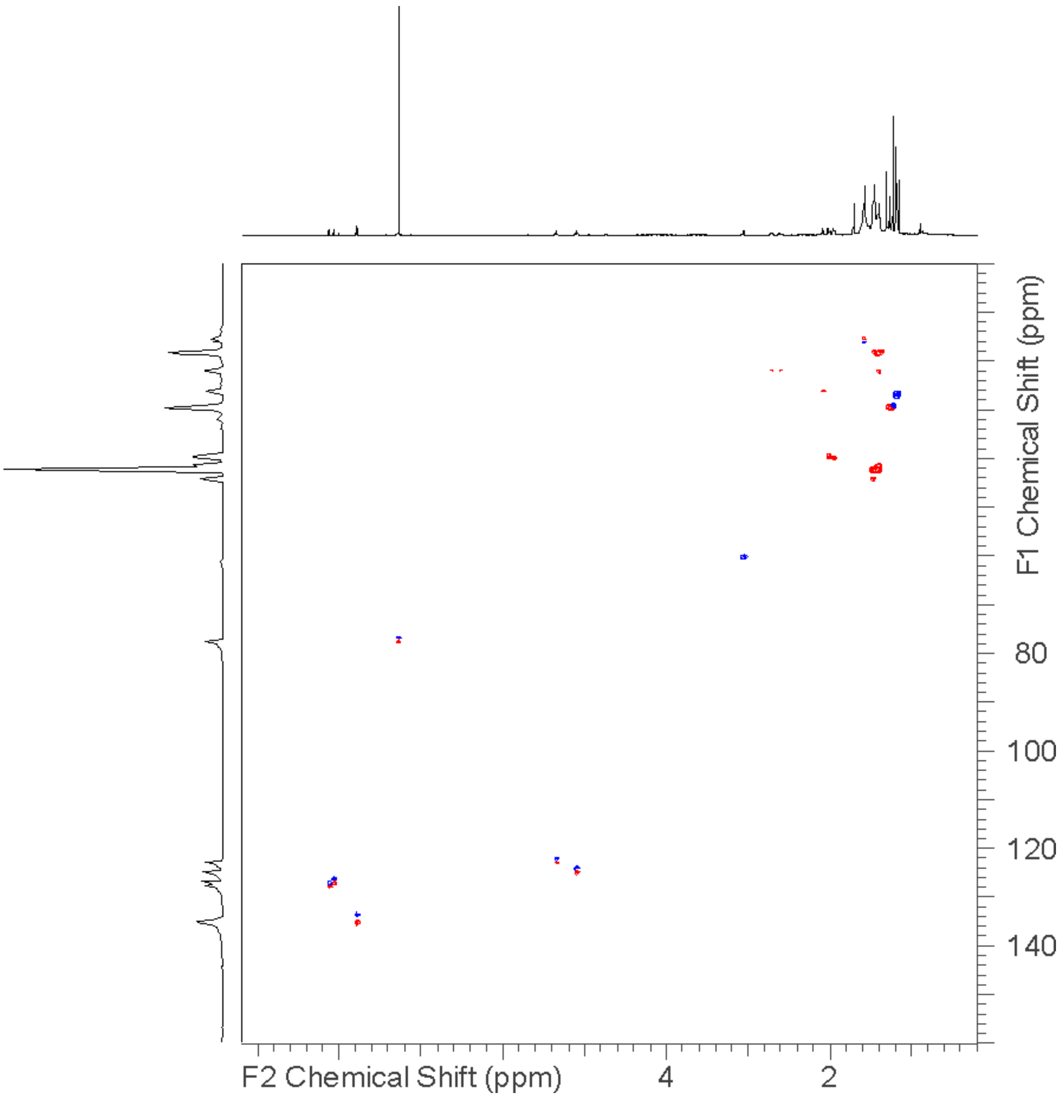

Figure S11. *HSQC spectrum of myxoquinone 843 acquired in CDCL₃ at 700/175 MHz*

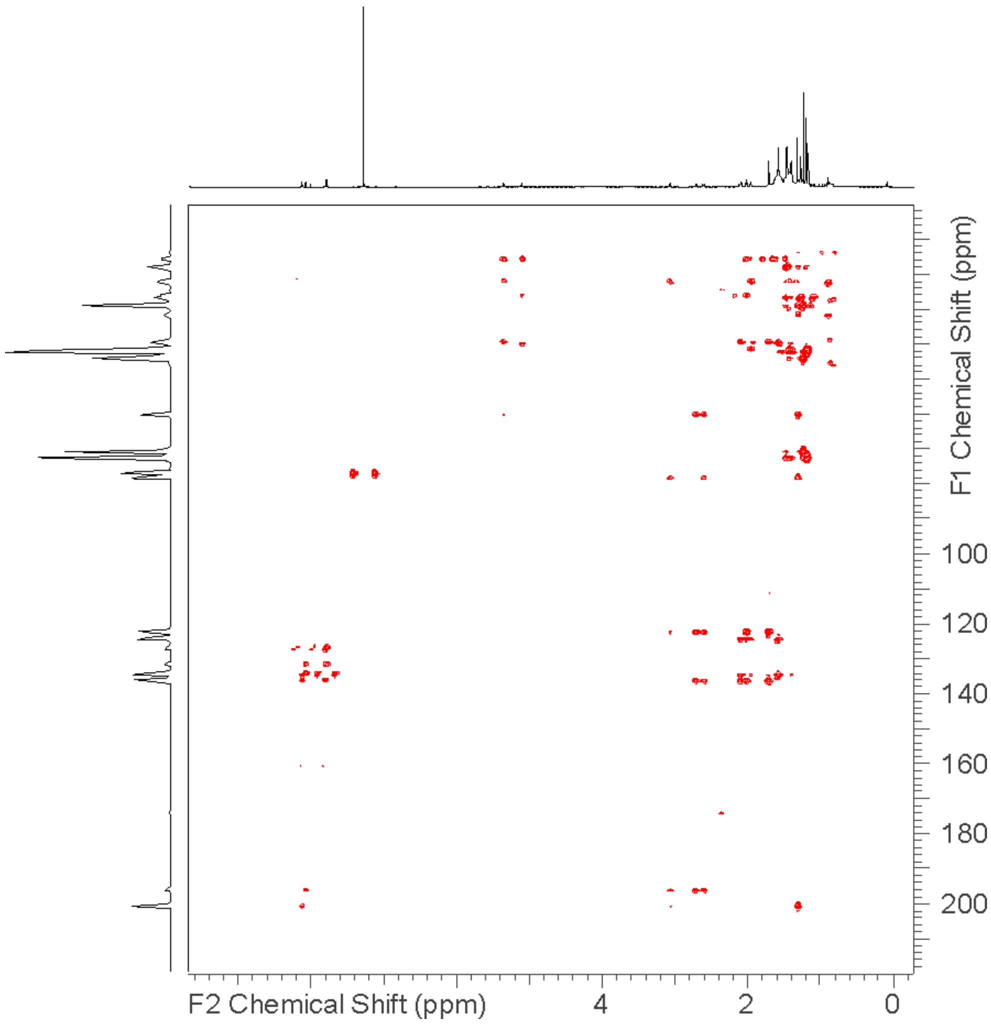

Figure S12. *HMBC spectrum of myxoquinone 843 acquired in $CDCl_3$ at 700/175 MHz*

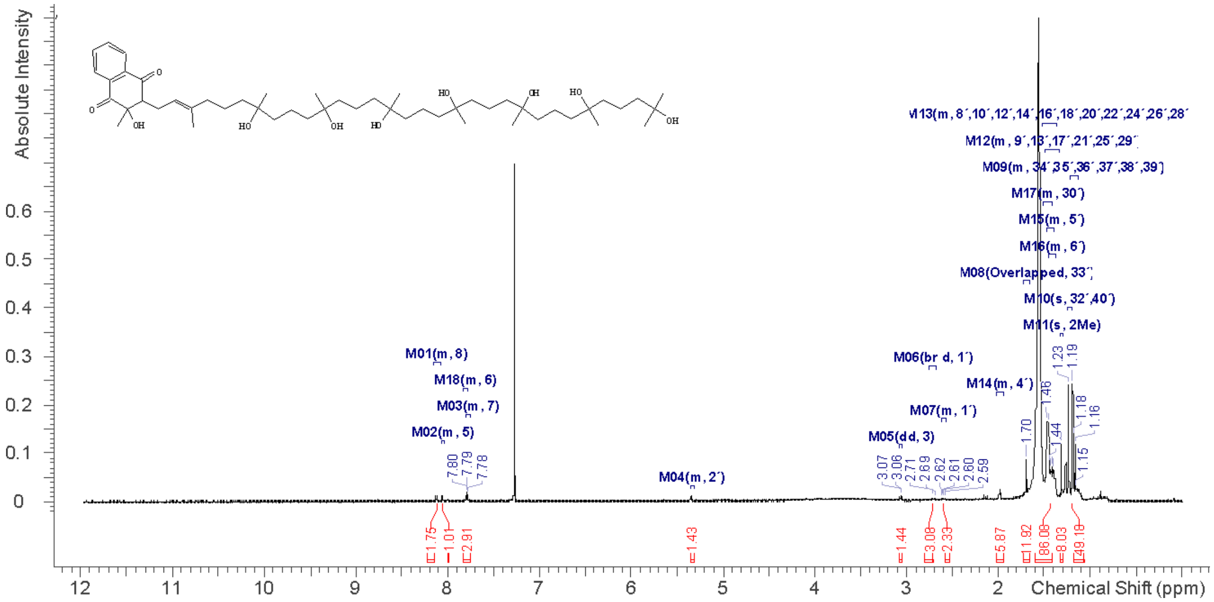

Figure S13. *^{1}H spectrum of myxoquinone 861 acquired in* $CDCl_3$ *at 700 MHz*

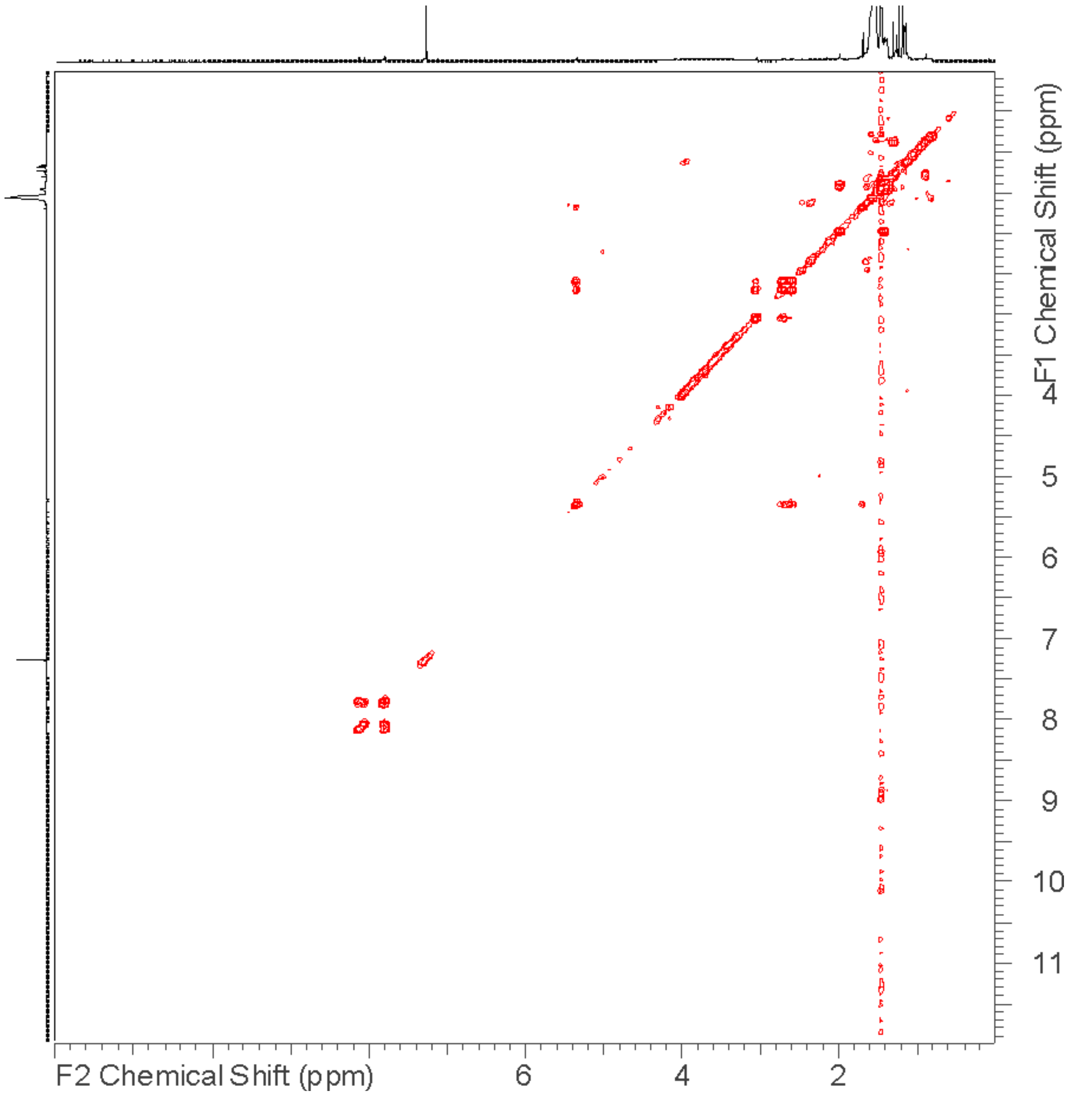

Figure S14. *COSY spectrum of myxoquinone 861 acquired in $CDCl_3$ at 700 MHz*

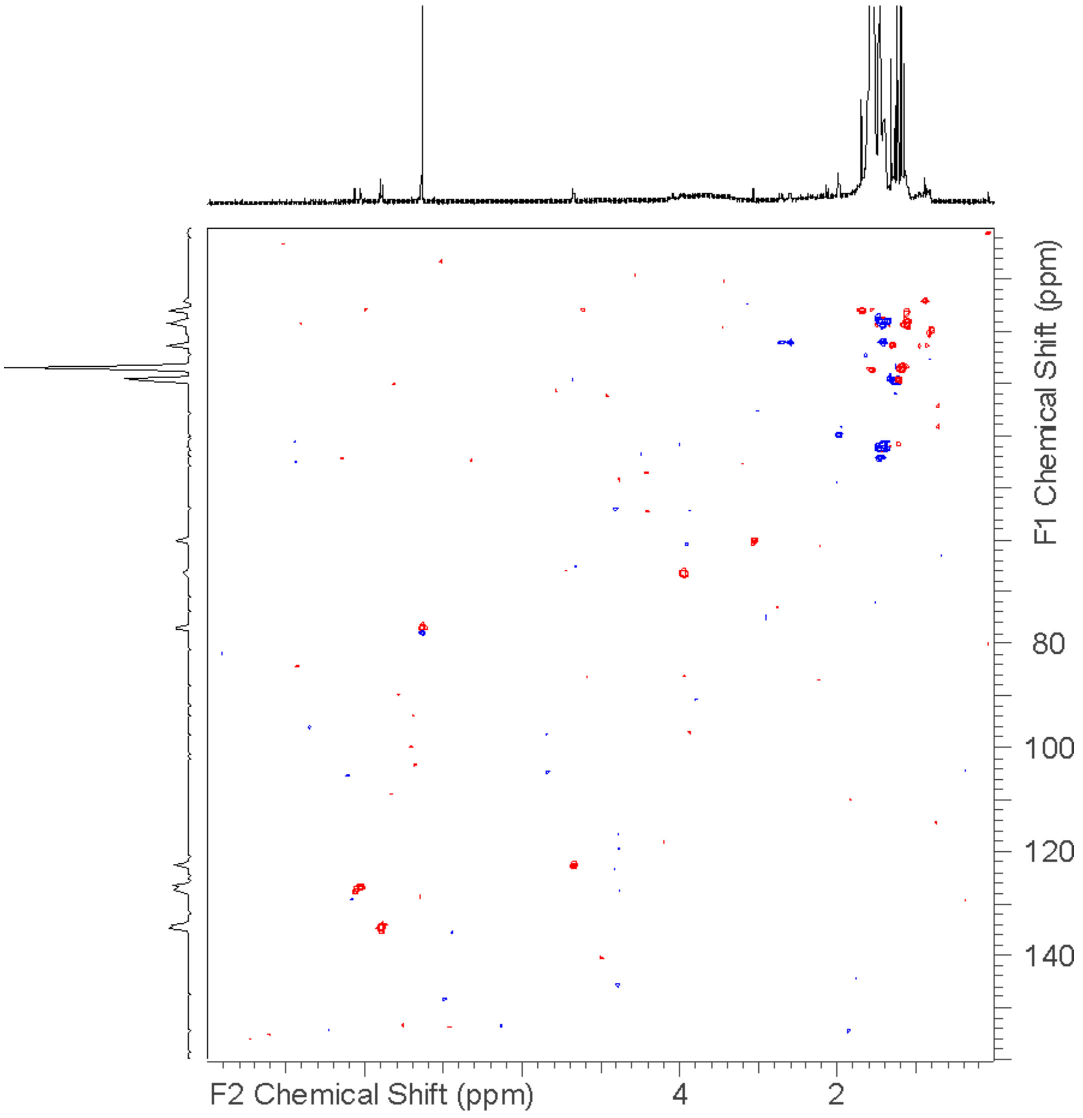

Figure S15. *HSQC spectrum of myxoquinone 861 acquired in $CDCl_3$ at 700/175 MHz*

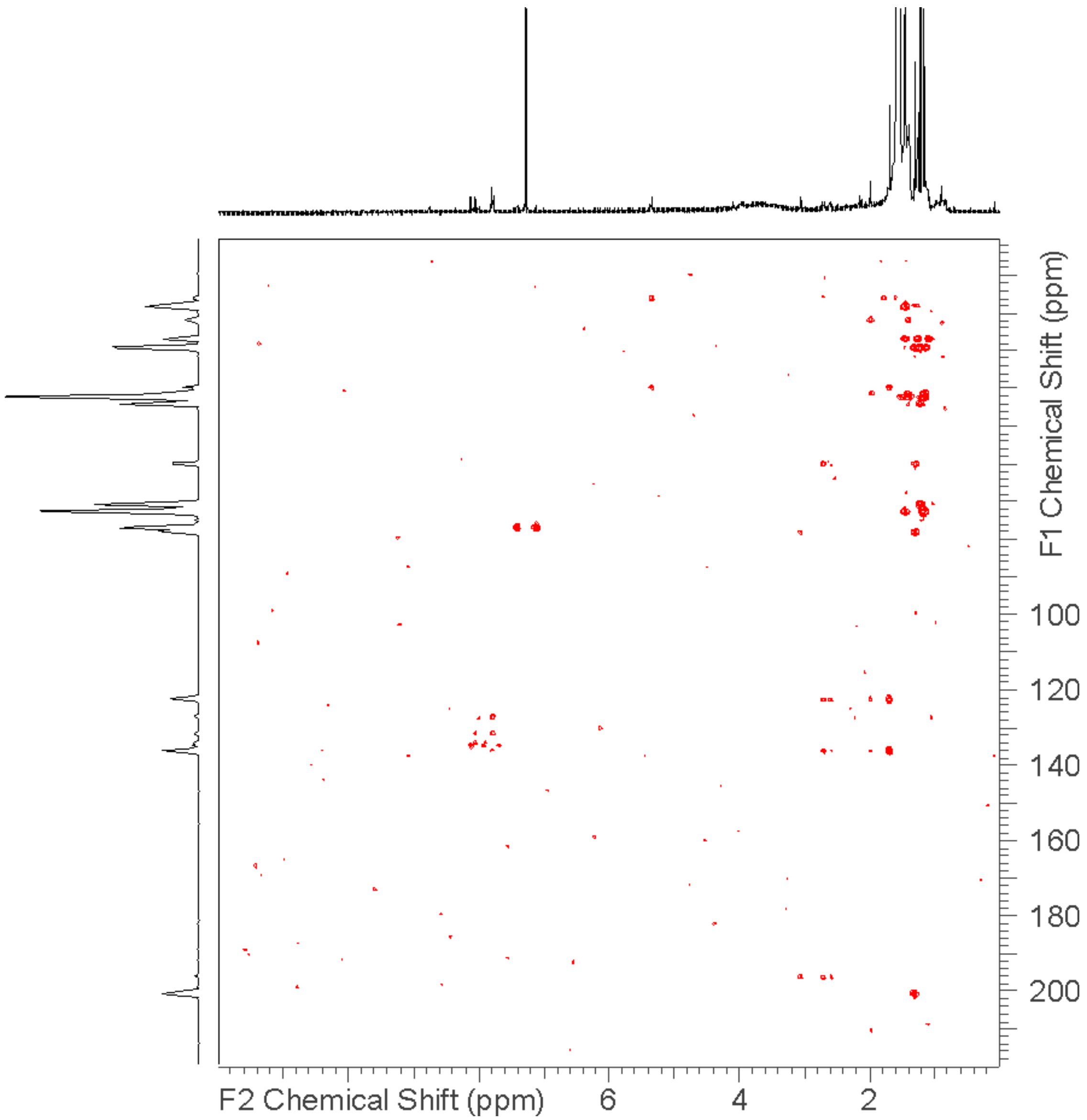

Figure S16. *HMBC spectrum of myxoquinone 861 acquired in $CDCl_3$ at 700/175 MHz*

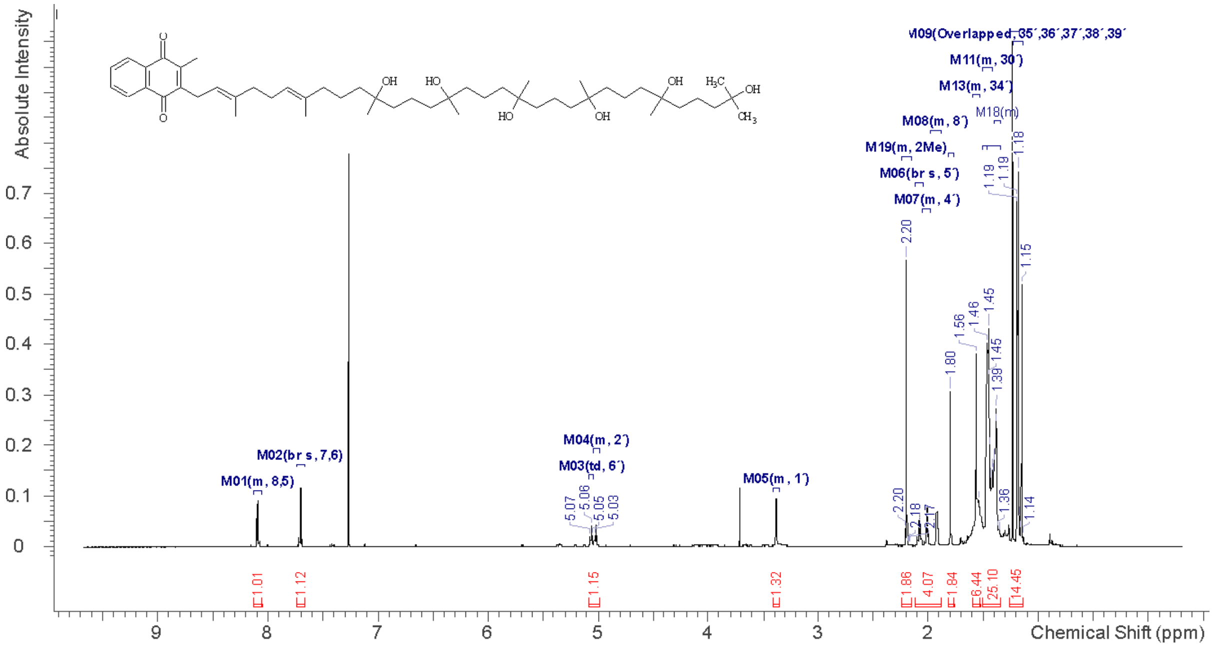

Figure S17. *1H spectrum of myxoquinone 825 acquired in CDCl3 at 700 MHz*

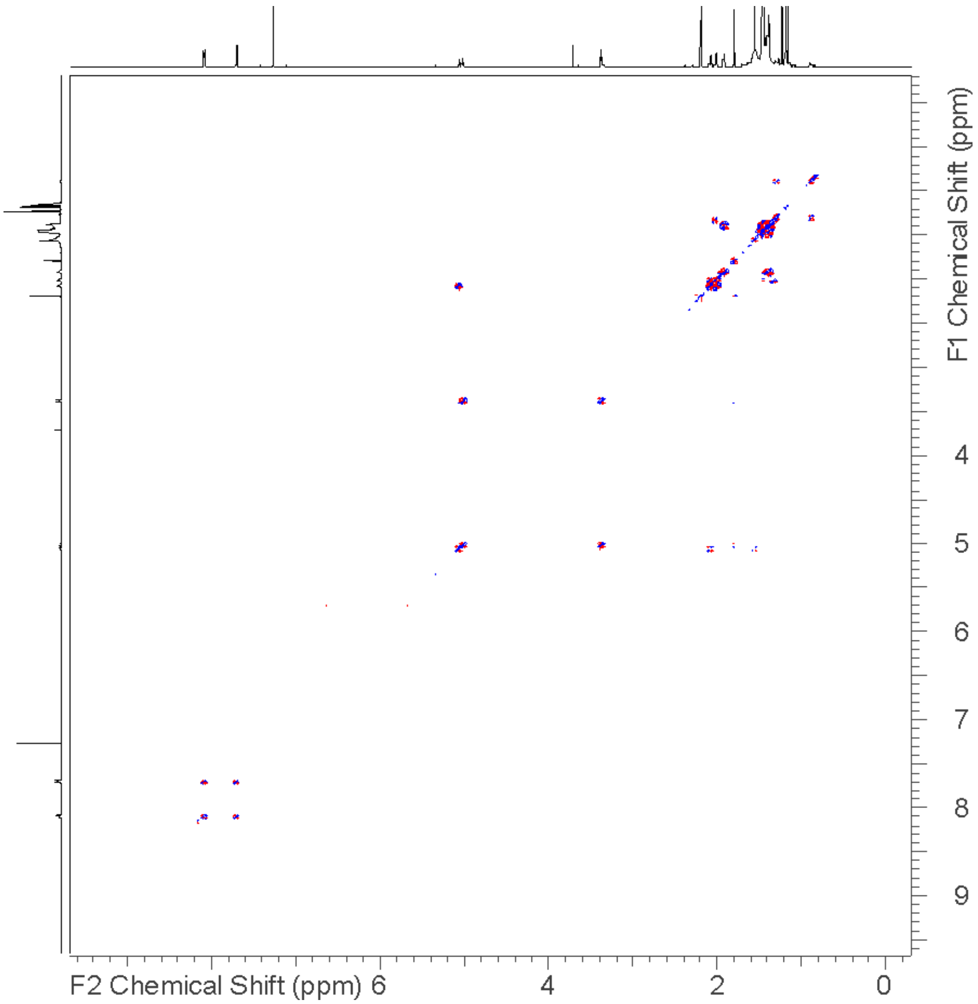

Figure S18. *DQF COSY spectrum of myxoquinone 825 acquired in $CDCl_3$ at 700/175 MHz*

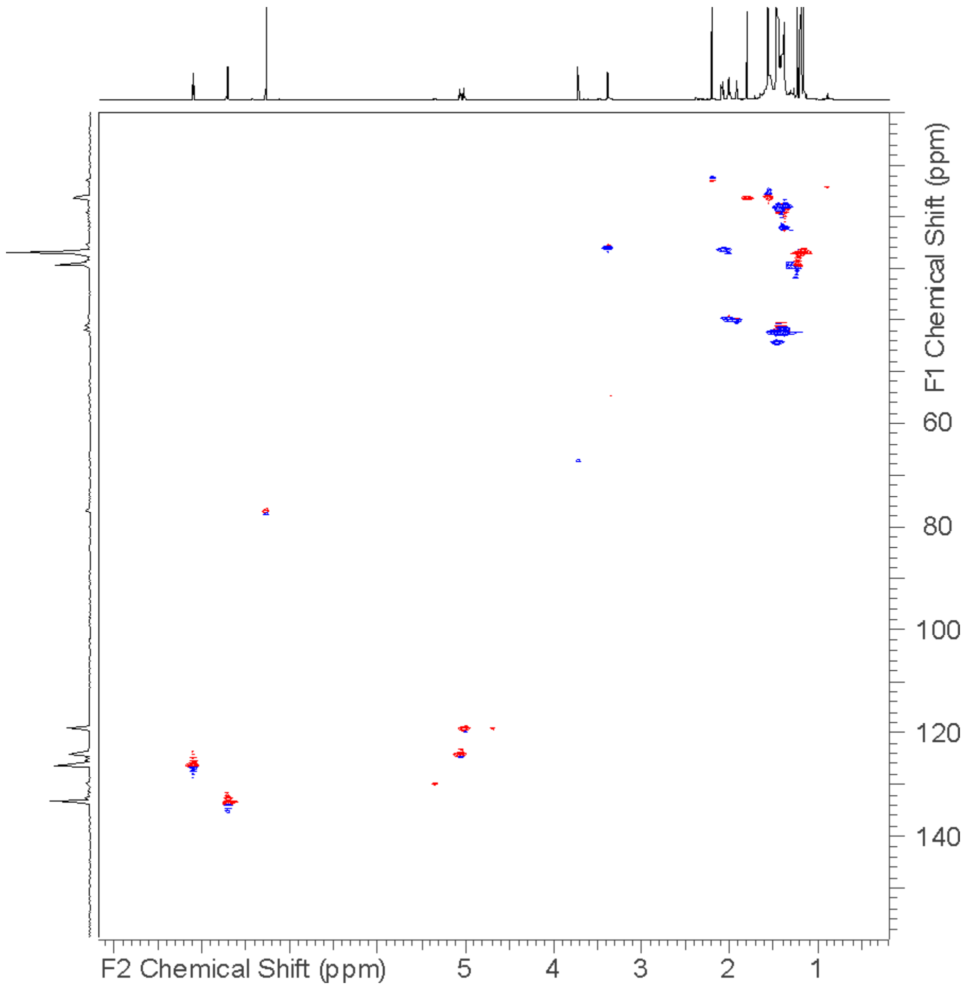

Figure S19. *HSQC spectrum of myxoquinone 825 acquired in $CDCl_3$ at 700/175 MHz*

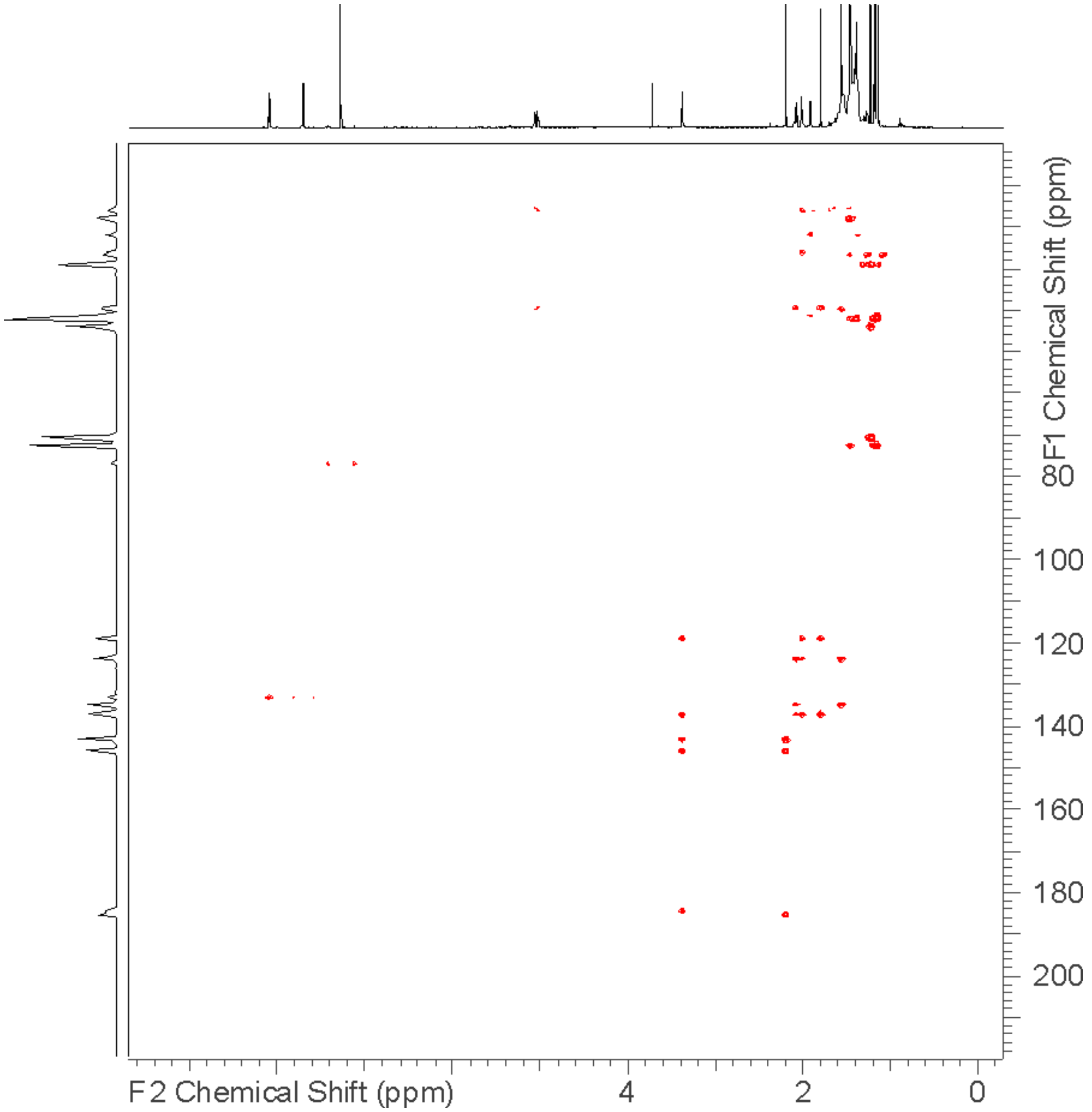

Figure S20. *HMBC spectrum of myxoquinone 825 acquired in $CDCl_3$ at 700/175 MHz*

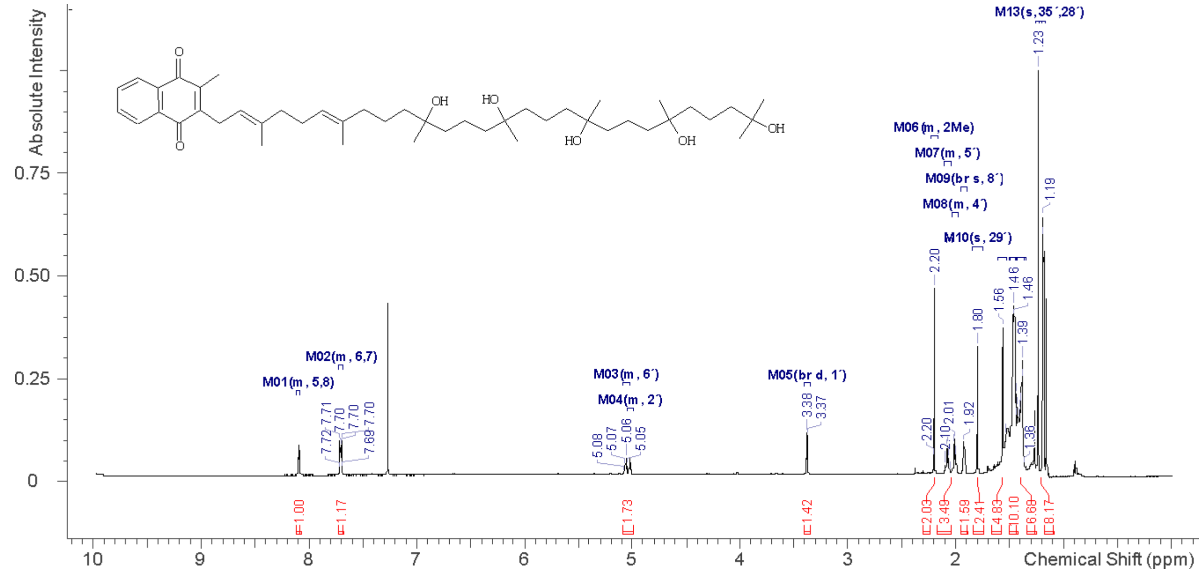

Figure S21. *^{1}H spectrum of myxoquinone 739 acquired in CDCl₃ at 700 MHz*

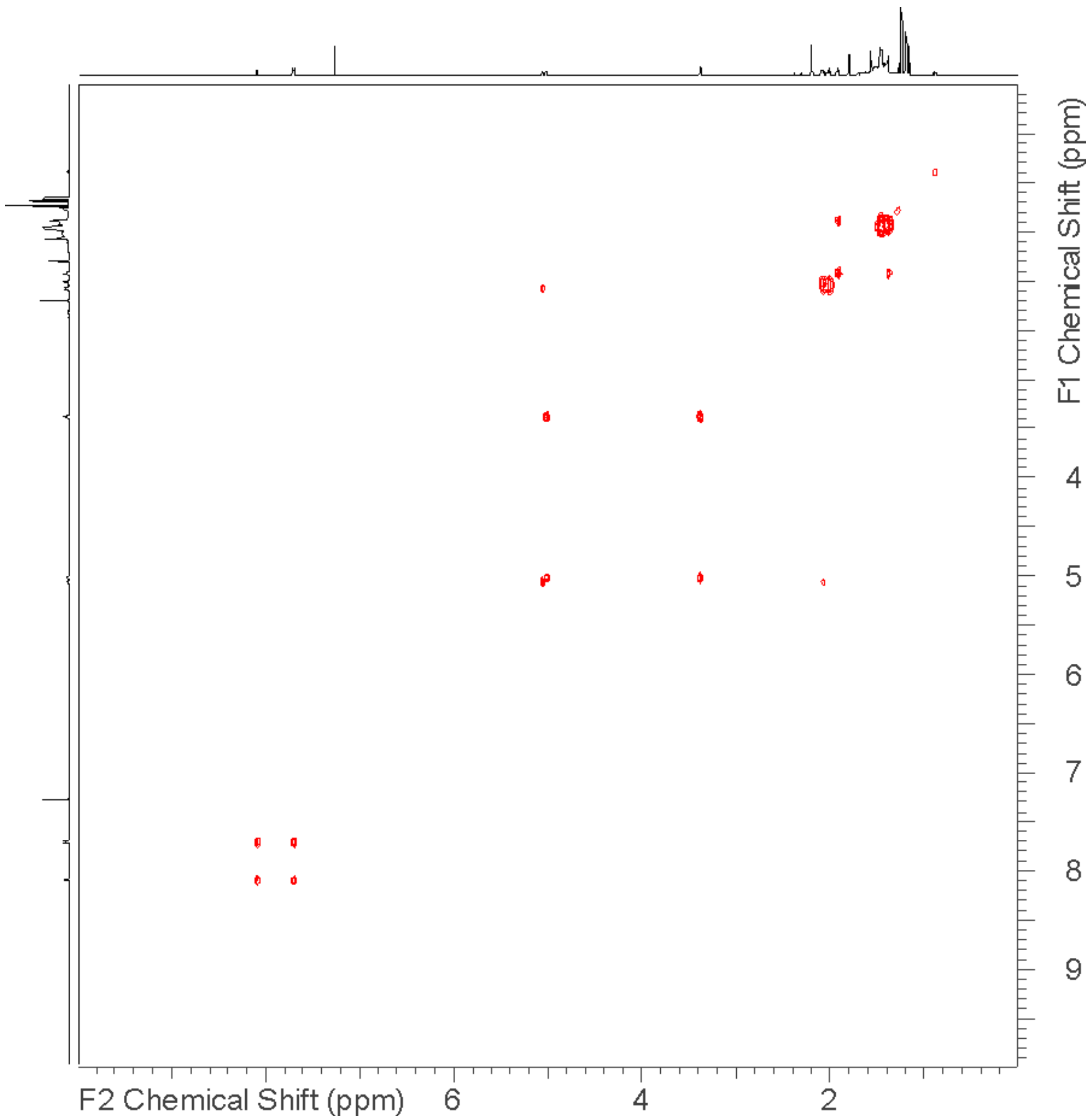

Figure S22. *COSY spectrum of myxoquinone 739 acquired in $CDCl_3$ at 700 MHz*

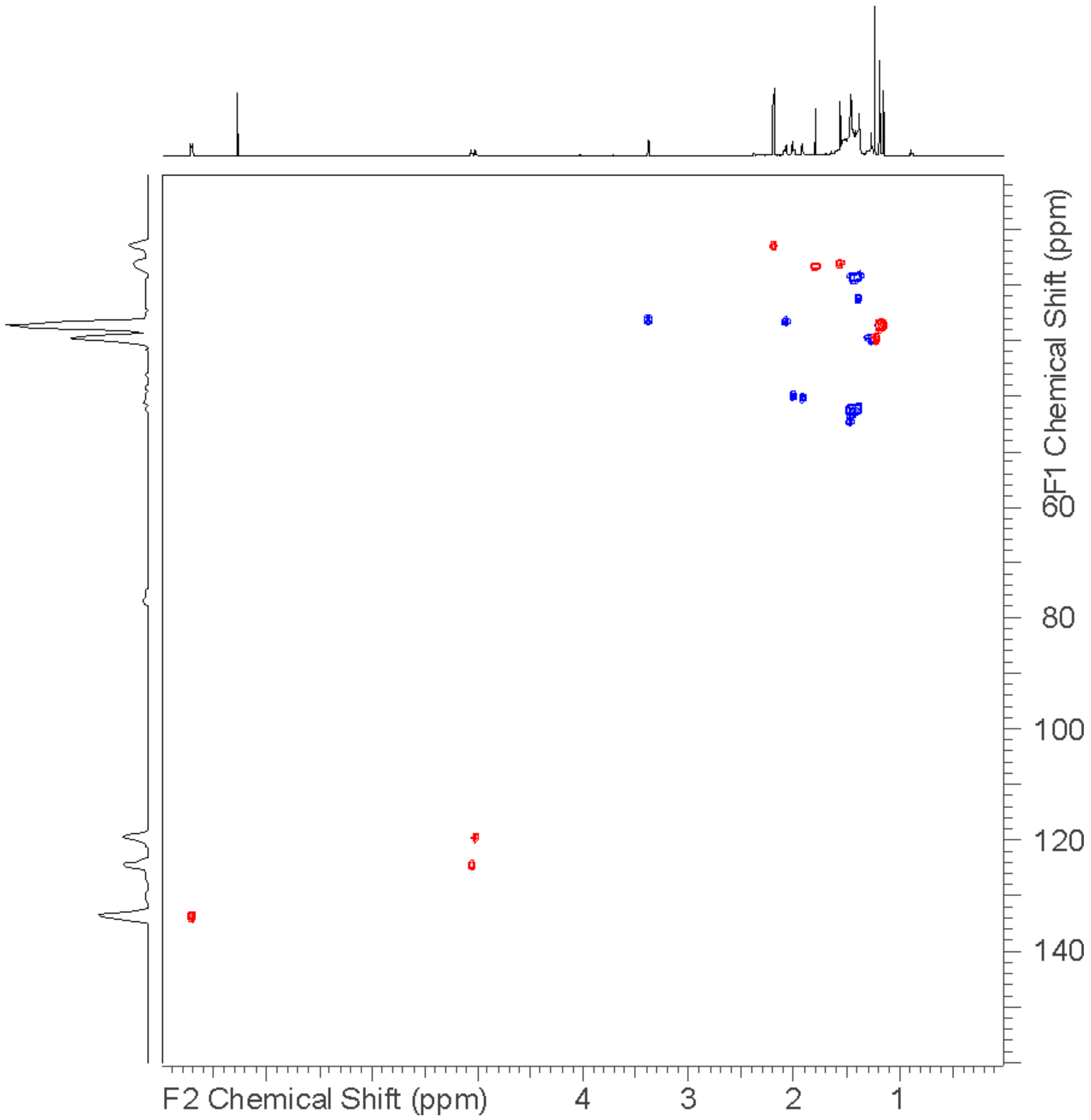

Figure S23. *HSQC spectrum of myxoquinone 739 acquired in $CDCl_3$ at 700/175 MHz*

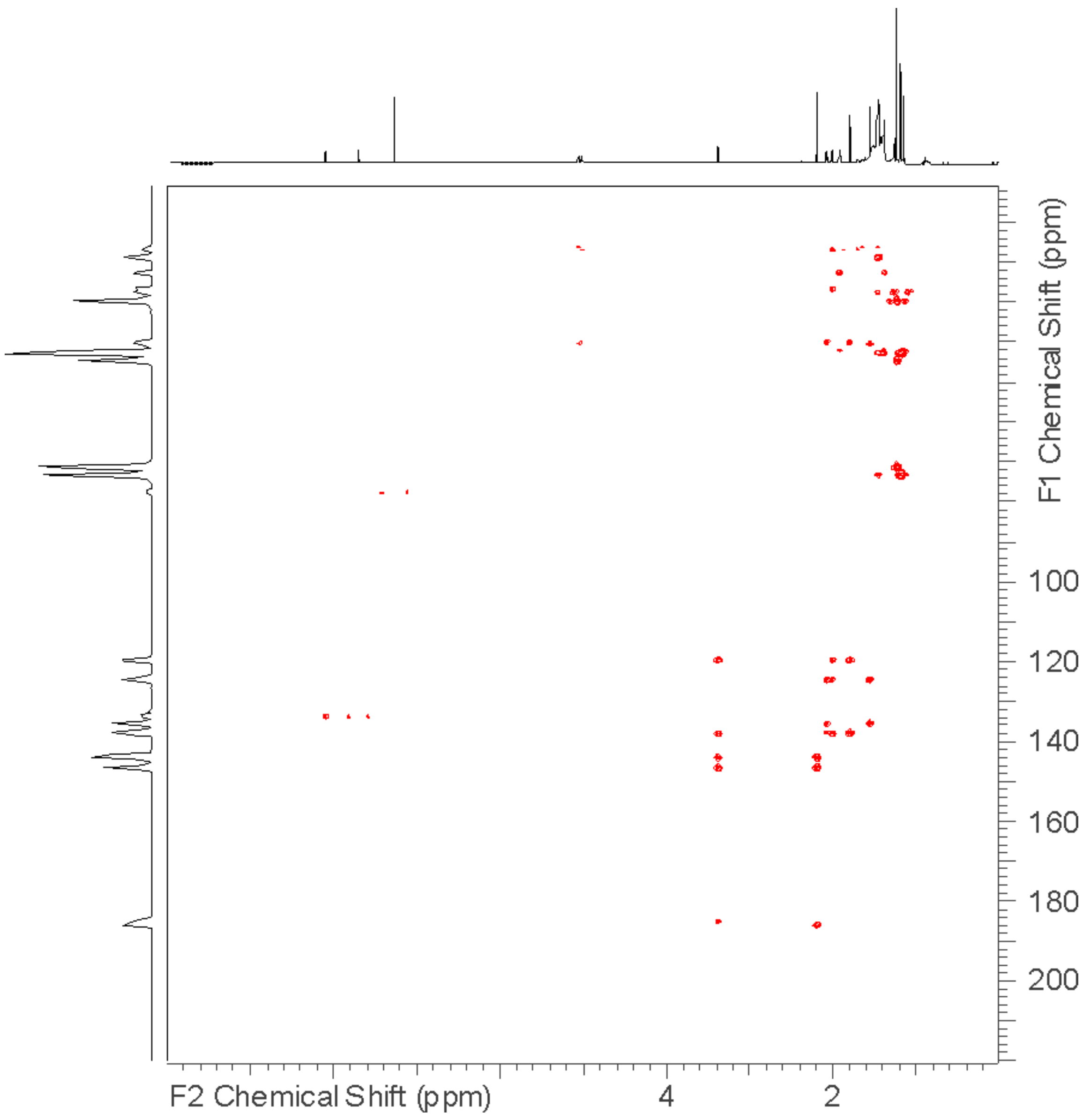

Figure S24. *HMBC spectrum of myxoquinone 739 acquired in $CDCl_3$ at 700/175 MHz*

6 Biological activity assays

Human HCT-116 colon carcinoma cells (ACC-581) were received from the German Collection of Microorganisms and Cell Cultures (Deutsche Sammlung für Mikroorganismen und Zellkulturen, DSMZ) and were cultured under the conditions recommended by the depositor. To determine the cytotoxic activities of the myxoquinones, cells from actively growing cultures were harvested and seeded at 5 x 10^4 cells per well in a 96 CELLBind® surface well plate in 120 µL 90% modified McCoy's 5A medium with 10% h. i. fetal bovine serum (FBS). After 2 h of equilibration, the cells were treated with the compounds in a serial dilution. After 5 days of incubation at 37 °C, 20 µL of 5 mg/mL thiazolyl blue tetrazolium bromide (MTT) in PBS was added. After discarding the medium, 100 µL of a 2-propanol 10 N HCl mixture (250:1) was added to dissolve formazan granules. A microplate reader (EL808, Bio-Tek Instruments Inc.) was used to determine the absorbance at 570 nm.

All microorganisms used for the biological assays were obtained from the German Collection of Microorganisms and Cell Cultures (Deutsche Sammlung für Mikroorganismen und Zellkulturen, DSMZ) or were part of our in-house strain collection and were cultured under the conditions recommended by the depositor. Bacterial cultures were prepared in MHB (2.9 g/L beef infusion solids, 17.5 g/L casein hydrolysate, 1.5 g/L starch at pH 7.4), M7H9 (0.5 g/L ammonium sulfate, 2.5 g/L disodium phosphate, 1.0 g/L monopotassium phosphate, 0.1 g/L sodium citrate, 0.05 g/L magnesium sulfate, 0.0005 g/L calcium chloride, 0.001 g/L zinc sulfate, 0.001 g/L copper sulfate, 0.04 g/L ferric ammonium citrate, 0.50 g/L *L*-glutamic acid, 0.001 g/L pyridoxine, 0.0005 g/L biotin at pH 6.6) or Myc 2.0 medium inoculated from the strain grown on agar plate. The compounds were diluted serially in sterile 96 well-plates before adding the bacterial cell suspension. The bacteria were grown for 24 h at RT, 30 °C or 37 °C. Growth inhibition was inspected visually. MIC50 values were determined relative to the respective control samples by sigmoidal curve fitting. Positive controls used for the respective microbial test strains are listed in Table S 11.

Table S11. *Microbial test strains and positive controls used for MIC determination.*

Microbial strain	Control
C. albicans	Amphotericin B
P. anomala	Amphotericin B
C. freundii DSM 30039	Ciprofloxacin-HCl
A. baumanii DSM 30008	Ciprofloxacin-HCl
S. aureus Newman	Vancomycin
B. subtilis DSM 10	Vancomycin
E. coli BW25113	Ciprofloxacin-HCl
P. aeruginosa PA14	Ciprofloxacin-HCl
M. smegmatis mc^2 155	Rifampicin

7 References

1. Garcia, R.O.; Krug, D.; Müller, R. Chapter 3. Discovering natural products from myxobacteria with emphasis on rare producer strains in combination with improved analytical methods. *Complex Enzymes in Microbial Natural Product Biosynthesis, Part B: Polyketides, Aminocoumarins and Carbohydrates* **2009**, *458*, 59–91, doi:10.1016/S0076-6879(09)04803-4.

2. Krug, D.; Zurek, G.; Schneider, B.; Garcia, R.; Müller, R. Efficient mining of myxobacterial metabolite profiles enabled by liquid chromatography-electrospray ionization-time-of-flight mass spectrometry and compound-based principal component analysis. *Anal. Chim. Acta* **2008**, *624*, 97–106, doi:10.1016/j.aca.2008.06.036.

3. Panter, F.; Krug, D.; Müller, R. Novel Methoxymethacrylate Natural Products Uncovered by Statistics-Based Mining of the *Myxococcus fulvus* Secondary Metabolome. *ACS Chem. Biol.* **2019**, *14*, 88–98, doi:10.1021/acschembio.8b00948.

4. Hoffmann, T.; Krug, D.; Bozkurt, N.; Duddela, S.; Jansen, R.; Garcia, R.; Gerth, K.; Steinmetz, H.; Müller, R. Correlating chemical diversity with taxonomic distance for discovery of natural products in myxobacteria. *Nat. Commun.* **2018**, *9*, 803, doi:10.1038/s41467-018-03184-1.

5. O'Leary, N.A.; Wright, M.W.; Brister, J.R.; Ciufo, S.; Haddad, D.; McVeigh, R.; Rajput, B.; Robbertse, B.; Smith-White, B.; Ako-Adjei, D.; et al. Reference sequence (RefSeq) database at NCBI: current status, taxonomic expansion, and functional annotation. *Nucleic Acids Res.* **2016**, *44*, D733-45, doi:10.1093/nar/gkv1189.

6. Gilchrist, C.L.M.; Chooi, Y.-H. Clinker & clustermap.js: Automatic generation of gene cluster comparison figures. *Bioinformatics* **2021**, doi:10.1093/bioinformatics/btab007.

7. Linington, R.G. npatlas - The Natural Products Atlas. Available online: https://www.npatlas.org.

8. van Santen, J.A.; Jacob, G.; Singh, A.L.; Aniebok, V.; Balunas, M.J.; Bunsko, D.; Neto, F.C.; Castaño-Espriu, L.; Chang, C.; Clark, T.N.; et al. The Natural Products Atlas: An Open Access Knowledge Base for Microbial Natural Products Discovery. *ACS Cent. Sci.* **2019**, doi:10.1021/acscentsci.9b00806.
